# Recent progress in the study of exosomes in the gastric cancer immune microenvironment

**DOI:** 10.3389/fimmu.2025.1595124

**Published:** 2025-07-23

**Authors:** Haibo Liu, Lei Zhu, Jianmei Yin

**Affiliations:** ^1^ Emergency Department, The First Hospital of Jilin University, Changchun, Jilin, China; ^2^ Department of Otolaryngology-Head and Neck Surgery, The First Hospital of Jilin University, Changchun, Jilin, China

**Keywords:** gastric cancer, exosomes, immunity, tumor microenvironment, tumor immunotherapy

## Abstract

Gastric cancer (GC) ranks among the most prevalent forms of cancer and contributes significantly to cancer-related mortality. There exists a pressing need to investigate novel approaches for GC management to improve diagnostic methods, therapeutic interventions, and patient outcomes. Exosomes are nanoscale extracellular vesicles (EVs) derived from various cell types that carry a diverse range of biomolecular cargo, including DNA, RNA, proteins, lipids, and other bioactive constituents. They play significant roles in GC pathogenesis and tumor microenvironment (TME) modulation. Exosomes derived from cancer cells can enhance tumor progression, transform the TME, and modulate immune responses. Immune cell-derived exosomes can similarly modulate immune functions and the TME. Immunotherapy represents a GC treatment breakthrough and is expected to show efficacy when combined with exosome-targeted therapy. Abundant research has demonstrated that exosomes are crucial for tumor growth, immune evasion, immune microenvironment reconfiguration, and immunotherapy efficacy in GC. This review describes the role of exosomes in the GC microenvironment, focusing on the mechanisms by which exosomes regulate immune responses to GC, and summarizes the current status of and challenges in the development of exosome-based diagnostics and immunotherapy for GC.

## 1 Introduction

Gastric cancer (GC) is the fourth-most common cause of cancer-related deaths and the fifth-most common cancer type globally (1). Factors affecting the development of GC include genetic polymorphisms, environmental exposures, age, sex, and infection with *Helicobacter pylori* (2). Due to the low proportion of early-stage diagnoses and lack of definite clinical symptoms, GC is commonly detected in the advanced metastatic phase, where the 5-year survival rate is only approximately 32% (3). Effective treatment options for GC include surgery, radiotherapy, chemotherapy, targeted therapy, and immunotherapy, but even after surgery, approximately 60% of patients experience local recurrence or distant metastasis (4). As a result, the exploration of new, robust biomarkers and therapeutic strategies is crucial for improving the prognosis and quality of life of GC patients.

The prognosis of GC patients and their responses to immunotherapy are impacted considerably by morphological and molecular heterogeneity in the tumor microenvironment (TME). Immunomodulatory cells in the GC TME include regulatory T cells, tumor-infiltrating myeloid-derived suppressor cells (MDSCs), tumor-associated macrophages (TAMs), and natural killer (NK) cells (5). Immune cells can also interact with cancer cells to influence the onset and progression of cancer.

**Figure 1:**
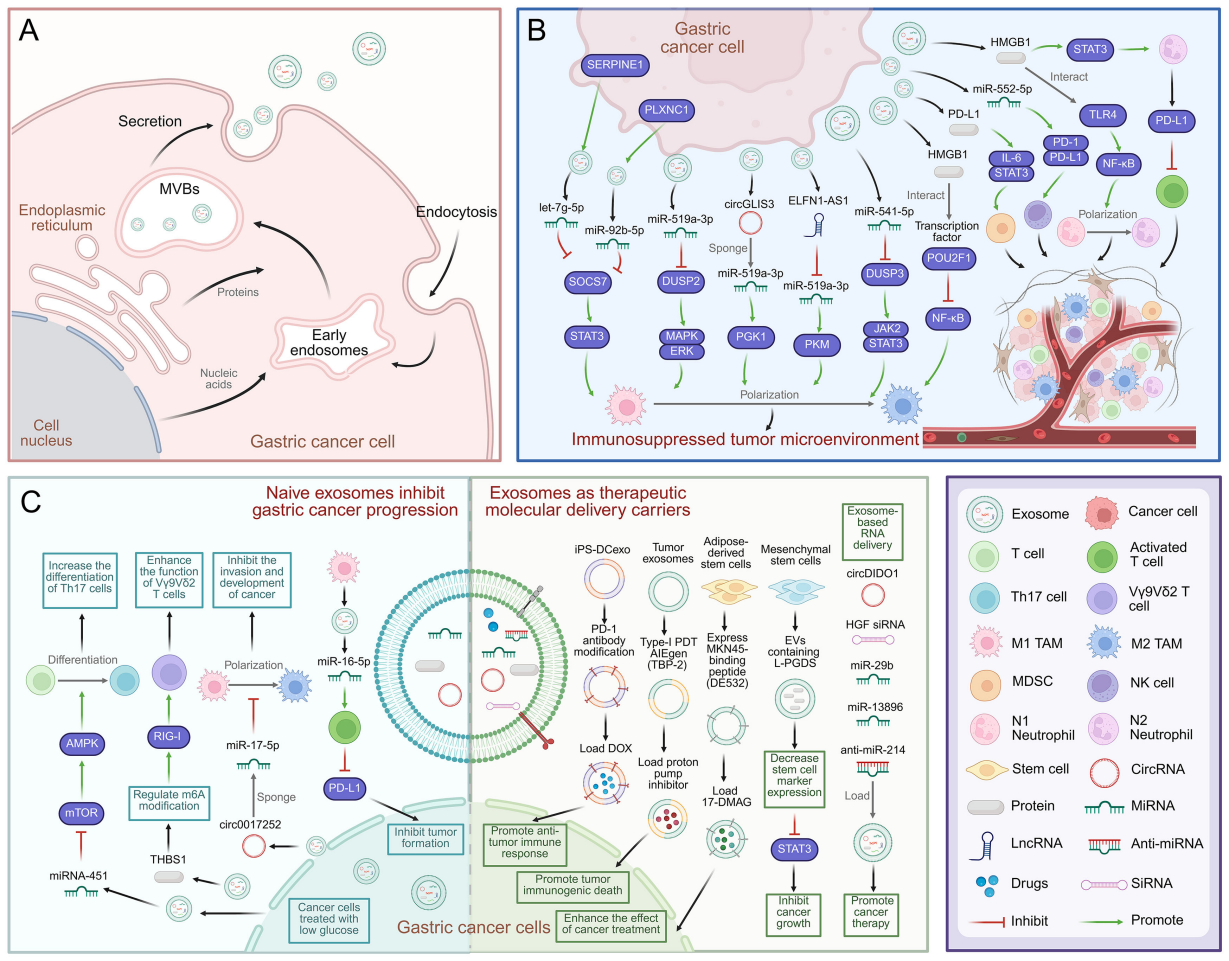
Mechanisms of exosome-mediated immune escape and immunotherapy in GC. **(A)** Schematic diagram of exosome biogenesis in GC cells. **(B)** Exosomes from GC mediate communication between GC cells and immune cells, and promote GC cell development and immunosuppression in immune TME. **(C)** Mechanism of natural exosomes promoting immunotherapy in gastric cancer. Exosomes can be used as carriers to deliver therapeutic molecules to promote the treatment of gastric cancer, including chemotherapy drugs, proteins and RNA. Created with BioRender.com.

Exosomes are a type of extracellular vesicle (EV) with diameters of 30–150 nm. They are secreted by virtually all types of cells and can be stably present in a variety of biological fluids (6). The biogenesis of exosomes commences with the invagination of the plasma membrane, a process that gives rise to early endosomes. These early endosomes subsequently mature into multivesicular bodies (MVBs), which fuse with the plasma membrane and are released into the extracellular milieu as exosomes (7) (**Figure 1A**). Under strict cellular regulation, released exosomes encapsulate a diverse array of biomolecules, including proteins, RNA, DNA, lipids, and cytokines (8). Exosomes are composed of a lipid bilayer that contains transmembrane proteins; delivery of this cargo can mediate local and distant cell communication under physiological and pathological conditions (9). Several studies have shown that exosome-mediated transport of bioactive signaling molecules in the TME has diagnostic and therapeutic functions (10). Indeed, numerous lines of investigation have demonstrated that the delivery of exosome cargo is vital for GC proliferation, metastasis, drug resistance, immune response, and treatment (11). Exosomes have also attracted attention in the field because they are more stable than circulating proteins and hormones and can serve as early biomarkers for cancer detection and disease progression (12). In addition, improvements in exosome engineering technology have led to the development of targeted exosomes, a promising way to deliver anti-neoplastic therapies (13). Indeed, due to their biocompatibility, low immunogenicity, and ability to transport biomolecules between cells and cross biological barriers, they are suitable for the targeted delivery of various therapeutic modalities such as small molecules, siRNA, and miRNA (14).

The tumorigenesis and progression of GC are intimately associated with immune cells and other types of mesenchymal stromal cells (MSCs), cytokines, and exosomes in the TME (15). Exosomes derived from cancer cells or tumor-associated immune cells can carry factors that suppress immune cell activity and help tumors evade immune surveillance (8). Therefore, it is important to investigate the mechanisms by which exosomes regulate the immune microenvironment of GC. To this end, this review aims to summarize the mechanisms of exosome-mediated GC development, focusing on recent studies of exosome-mediated immune escape. In addition, tumor immunotherapies, which consist of immune checkpoint inhibitors, cellular immunotherapy, and therapeutic cancer vaccines, have drawn substantial attention within the field. Identifying key targets and elucidating the molecular mechanisms associated with GC immunity will improve our understanding of GC pathogenesis and augment immunotherapy efficacy (16). Thus, we have reviewed the role of exosomes in GC immune responses, as well as recent advances in exosome-targeted treatments. Together, these studies provide a basis for potential GC treatment strategies.

## 2 Exosomes in GC diagnosis and prognosis

Abundant evidence indicates that exosomes are intricately linked to GC tumorigenesis, progression, metastasis, immune escape, and drug resistance through the delivery of functional biomolecules (17). Cancer initiation and development are generally influenced by the function of cancer cells or cells in the microenvironment. The bioactive substances carried by exosomes, including exosomal proteins, miRNAs, long non-coding RNAs (lncRNAs), and circular RNAs (circRNAs), are involved in many important processes (18) as described below, including the diagnosis, prognosis, and clinical treatment of GC (**Table 1**).

**Table 1:** Exosomes in GC diagnosis and prognosis.

Origin	Target	Contents	Mechanism	Effect	Ref.
GC cells	Circulating EVs	LncGC1	As an early marker of therapeutic efficacy of neoCT		(25)
GC cells	Serum	PD-L1	Reflect the immunosuppressive state of advanced GC patients		(26)
GC cells	Plasma	PKM2	Plasma exosome PKM2 is a novel biomarker and can be used for the clinical diagnosis of GC		(27)
GC cells	Plasma	Circ_0079439	Exosomal circ_0079439 may be a potential biomarker for the early and late diagnosis of GC		(28)
GC cells	Serum	LncHOTTIP	As a potential diagnostic and prognostic biomarker for gastric cancer		(29)
GC cells	Peritoneal lavage samples	Let-7g-3p and miR-10395-3p	Can be used as a biomarker to predict the effect of peritoneal metastasis and systemic chemotherapy		(30)
GC cells	GC cells	GRP78	Promote cancer stemness		(31)
GC cells	Lymphatic endothelial cells	LncAKR1C2	Exosomal lncAKR1C2 promotes CPT1A expression by regulating YAP phosphorylation	Enhance tube formation and migration of lymphatic endothelial cells, and promote lymphangiogenesis and lymphatic metastasis *in vivo*	(33)
GC cells	Human umbilical vein endothelial cells	NOS3	MiR-605-3p mediates the production of exosomal NOS3	Induce angiogenesis, establishe liver PMN, and promote liver metastasis	(35)
GC cells	Serum	CircHIPK3	CircHIPK3 blocks autophagy dependent ferroptosis	Can be used as a noninvasive index to evaluate cisplatin resistance in GC	(38)
Cisplatin-resistant GC cells	GC cells	MiR-769-5p	Exosomal miR-769-5p targets caspase-9 and promote ubiquitination degradation of p53	Lead to cisplatin resistance in GC and promote cancer progression	(39)
Cisplatin-resistant GC cells	GC cells	Lnc00852	Exosomal lnc00852 regulates COMMD7 through miR-514a-5p	Lead to cisplatin resistance in GC	(40)
CAFs	GC cells	MiR-522	Exosomal miR-522 targets ALOX15 and inhibits ferroptosis	Mediate acquired chemotherapy resistance in GC	(42)

Early detection and diagnosis of GC are crucial for improving patient prognosis. Liquid biopsy is a non-invasive method for detecting circulating tumor cells, circulating tumor DNA, and EVs in serum and other bodily fluids (19). Research has employed AI to analyze surface-enhanced Raman spectra (SERS) of exosomes, leveraging classification models that recognize signal patterns in plasma exosomes to enable simultaneous identification of multiple early-stage cancers—including GC (20). Exosomal ncRNAs and proteins have shown great promise as molecular cancer diagnostic biomarkers (21). Indeed, 182 candidate GC biomarkers in serum exosomes have been identified via RNA sequencing, facilitating the machine learning-assisted identification of exosomal ncRNA characteristics for non-invasive, early detection of GC (22). The characteristics of miRNA in circulating exosomes can also predict peritoneal metastasis in patients with advanced gastric cancer (23). In addition, the characteristics of circulating exosome-derived mRNA, miRNA, and lncRNA in liquid biopsies have the potential to predict the therapeutic outcomes of neoadjuvant chemotherapy (neoCT) in advanced GC patients (24). Still other research has demonstrated that lncRNA-GC1 derived from circulating EVs serves as an early indicator of neoCT efficacy and can predict the survival rates of GC patients undergoing this therapy (25). Additionally, exosomal programmed death ligand 1 (PD-L1) is linked to systemic inflammatory markers, immunomodulatory cytokines, and T cells, and exosomal PD-L1 in serum may reflect an immunosuppressive state in advanced GC patients (26). Plasma-derived exosomal PKM2 modulates the TME by activating the SREBP1-associated lipid synthesis pathway in macrophages. Elevated expression of exosomal PKM2 correlates with unfavorable prognosis in GC patients and represents a promising novel biomarker (27). Another study result indicates that the upregulated plasma exosomal hsa_circ_0079439 in patients with GC may be a potential biomarker for the early and late diagnosis of GC (28). Besides, it has been demonstrated that exosomal-derived lncRNA HOTTIP may be a novel biomarker for GC diagnosis and outcome prediction (29). Other studies have shown that exosomes from GC peritoneal lavage fluid are rich in numerous miRNAs associated with peritoneal metastasis, such as let-7g-3p and miR-10395-3p, which can be used as biomarkers of peritoneal metastasis and chemotherapy efficacy (30). Moreover, studies have employed ultrasensitive ELISA combined with thio-NAD cycling to quantify trace amounts of 78-kDa glucose-regulated protein (GRP78) in exosomes released by GC cells. It was found that GRP78-enriched exosomes can promote cancer stemness (31).

Exosomes also have the capacity to facilitate the migration/metastasis of GC cells to local or distant tissues and organs. At initial diagnosis, over half of GC patients have detectable lymph node metastases, which frequently results in the development of distant metastasis and a poor prognosis (32). GC cell-derived exosomal lncAKR1C2 encodes the micro-protein pep-AKR1C2, which enhances CPT1A expression through the regulation of YES-associated protein 1 (YAP1) phosphorylation. It also improves the tube-forming ability and migratory capacity of lymphatic endothelial cells, thereby facilitating lymphangiogenesis and promoting tumor lymphatic metastasis *in vivo* (33). The establishment of pre-metastatic niches (PMN) in distant organs is critical for tumor metastasis (34). Studies have shown that nitric oxide synthase 3 (NOS3) originating from GC cell-derived exosomes can increase the concentration of nitric oxide within human umbilical vein endothelial cells, triggering angiogenesis, facilitating the formation of liver PMNs, and augmenting GC hepatic metastasis (35). Additionally, studies have isolated plasma exosomes from metastatic GC patients undergoing systemic chemotherapy and developed a liquid biopsy assay based on exo-miRNAs, and differentially expressed exo-miRNAs were identified as potential biomarkers for predicting chemotherapy resistance in GC patients (36).

Exosomes also mediate intercellular crosstalk during cancer progression and enhance therapeutic resistance. Currently, cisplatin-based chemotherapy is the primary treatment choice for advanced GC patients; however, a significant number of patients exhibit cisplatin resistance due to epigenetic alterations, signaling pathway aberrations, and disruptions in cell metabolism (37). CircHIPK3 has been shown to promote GC cisplatin resistance by obstructing ferroptosis and, when present in serum exosomes, may be a non-invasive marker of cisplatin resistance (38). In addition, exosomal miR-769-5p from cisplatin-resistant GC cell lines imparts cisplatin resistance to recipient GC cells. It further promotes cancer progression by targeting caspase-9 and enhancing the ubiquitination and degradation of p53 (39). Furthermore, exosomal lnc00852 originating from cisplatin-resistant GC cells also modulates COMM domain containing 7 (COMMD7) via miR-514a-5p to promote cisplatin resistance in recipient cells (40). Finally, cancer-associated fibroblasts (CAFs) are the primary stromal cell type in the TME (41). These cells secrete exosomal miR-522, which inhibits cancer cell ferroptosis by targeting arachidonate 15-lipoxygenase (ALOX15) and blocking lipid peroxidation, mediating acquired chemotherapy resistance in GC (42).

## 3 Exosomes can mediate GC immune escape

### 3.1 Exosomes derived from GC cells mediate immune escape

Cancer cells can regulate the immune milieu by releasing exosomes. Many studies have revealed that exosomes make critical contributions to the reconfiguration of the TME, thereby facilitating cancer cells’ escape from the immune system (43) (**Figure 1B**). Research has demonstrated that exosomes extracted from GC cell lines can alter the function and gene expression of CD8+ T cells, increase the frequency of effector memory CD4+ T cells and MDSCs, and reduce the frequency of CD8+ T cells and NK cells. Consistent with this observation, mice injected with GC cell-derived exosomes develop an immunosuppressive pulmonary TME. Studies have shown that exosomes originating from GC cells regulate the TME by suppressing immune function (44). Therefore, exploring the molecular characteristics of exosomes is critical for improving our understanding of immune escape mechanisms in GC (45).

T cell activation is central to the anti-tumor immune response (46). Some studies have found that exosomal circMAN1A2 can promote the development of GC and inhibit T cell anti-tumor activity. Specifically, circMAN1A2 competes with F-Box and WD repeat domain containing 11 (FBXW11) to bind and stabilize splicing factor proline and glutamine rich (SFPQ) expression, inhibiting T cell receptor stimulation and reducing T cell anti-tumor activity (47). Moreover, other studies have shown that lysine-specific demethylase 1 (LSD1) restricts T cell responses in the GC TME by triggering the aggregation of PD-L1 in GC exosomes, providing a novel target for GC immunotherapy (48). Finally, Vγ9Vδ2 T cells can effectively internalize exosomes derived from GC cells that carry miR-135b-5p. The miR-135b-5p impairs the function of Vγ9Vδ2 T cells by targeting specific protein 1 (SP1), inducing apoptosis, and reducing the production of the cytotoxic cytokines IFN-γ and TNF-α. Thus, targeting the exosomal miR-135b-5p/SP1 axis may improve the efficiency of Vγ9Vδ2 T cell-based GC immunotherapy (49).

TAMs, particularly M2-polarized TAMs, can be recruited and regulated by tumor-derived inflammatory cytokines and immunosuppressive metabolites, rendering them important mediators of GC tumor progression, immune escape, and therapeutic resistance (50). Studies have demonstrated that in GC cells, increased serpin family E member 1 (SERPINE1) expression leads to higher levels of let-7g-5p in exosomes. In turn, exosomal let-7g-5p is transferred to and taken up by macrophages, reducing the levels of suppressor of cytokine signaling 7 (SOCS7). By disrupting the interaction between SOCS7 and signal transducer and activator of transcription 3 (STAT3), it removes the inhibitory effect on STAT3 phosphorylation, leading to STAT3 over-activation and driving M2 polarization (51). Additionally, Biglycan belongs to the family of small proteoglycans. GC cell-derived exosomes containing Biglycan are delivered to macrophages, which triggers M2 polarization and upregulates CXCL10 expression. Consequently, this mechanism activates the JAK/STAT1 signaling pathway and enhances the proliferative, invasive, and metastatic capabilities of GC cells (52). Liver metastasis (LM) confers a poor prognosis to individuals suffering from GC. Notably, miR-519a-3p expression in exosomes from GC patients with LM is strikingly elevated compared to exosomes from GC patients without LM. Exosomal miR-519a-3p triggers the activation of the MAPK/ERK pathway by targeting dual specificity phosphatase 2 (DUSP2), resulting in the M2-like polarization of macrophages, facilitating the establishment of a pre-metastatic intrahepatic niche, and promoting GC-LM progression (53). In addition, plexin C1 (PLXNC1) inhibits SOCS7-STAT3 interactions by transferring GC cell-derived exosomal miR-92b-5p to macrophages, activating STAT3, and promoting GC cell proliferation and M2 TAM polarization (54). In addition, exosomal circGLIS3 promotes GC metastasis and the M2-like polarization of macrophages. Mechanistically, circGLIS3 sequesters miR-1343-3p, up-regulating PGK1 expression and modulating vimentin phosphorylation to drive GC tumorigenesis (55). Additionally, ELNF1-AS1 is highly enriched in GC-derived exosomes and targets miR-4644 to trigger pyruvate kinase M1/2 (PKM) expression. Exosomal ELNF1-AS1 in GC exosomes can also regulate glycolysis through PKM in a hypoxia inducible factor 1 subunit α (HIF-1α)-dependent manner, where it contributes to M2 TAM polarization and macrophage recruitment, thereby enhancing the growth and metastatic capacity of GC cells (56). Another study demonstrated that GC cells can also induce macrophage M2 polarization through the DUSP3/JAK2/STAT3 pathway, which is mediated by exosomal miR-541-5p (57). Moreover, the polarization of macrophages towards the M2-like phenotype is regulated by the deactivation of the NF-κB signaling pathway. This process results from the inhibition of p50 transcriptional activity via the engagement of high mobility group box-1 protein (HMGB1), present in exosomes derived from GC cells, with the transcription factor POU class 2 homeobox 1 (POU2F1) (58).

MDSCs are the principal immunosuppressive cells in the TME, and up-regulation of PD-L1 expression in the gastric epithelium can increase the number of tumor-infiltrating MDSCs (59). Studies have revealed that exosomal PD-L1 from GC cells may promote immunosuppression by promoting MDSC clustering and proliferation by activating the IL-6/STAT3 signaling pathway (60). Neutrophils are also important players in cancer development and progression and can promote cancer growth, metastasis, angiogenesis, and immunosuppression (61). Some studies have shown that neutrophils can promote tumor phenotypes through polarization. Specifically, exosomes derived from GC cells induce neutrophil autophagy and promote tumor activation through HMGB1/TLR4/NF-κB signaling (62). In addition, EVs in the GC microenvironment convey HMGB1 to trigger STAT3 activation, which up-regulates PD-L1 gene expression in neutrophils, thus inhibiting T cell-mediated immunity and highlighting the multidimensional role of EVs in regulating the immunosuppressive microenvironment (63). Furthermore, NK cells are crucial for immune homeostasis and preventing tumorigenesis; however, reductions in their efficiency have been noted in both GC tissue and peripheral blood (64). Indeed, studies have revealed that miR-552-5p derived from GC cell exosomes can drive the progression of GC by modulating the PD-1/PD-L1 axis, which affects NK cell function and impacts GC EMT (65).

### 3.2 Exosomes from immune cells mediate GC immune escape

Considerable evidence has shown that exosomes produced by immune cells can also impact the TME and are important regulators of tumor progression (**Supplementary Figure 1**). In particular, the multifunctional role of M2 TAM-derived exosomes in cancer progression has been extensively studied. Studies have shown that MALAT1 from M2 TAM-derived exosomes engages with the δ-catenin protein and impedes its ubiquitination and degradation via β-transducin repeats-containing proteins (β-TRCP). Moreover, MALAT1 sequesters miR-217-5p to up-regulate HIF-1α expression, thereby enhancing aerobic glycolysis in GC cells. These findings imply that M2 TAM-derived exosomes facilitate GC progression through MALAT1-mediated glycolytic regulation, presenting a potential target for GC treatment (66). Moreover, TAMs are a unique group of immune cells that express apolipoprotein E (ApoE) in the GC microenvironment. Indeed, M2 macrophage-derived exosomes trigger the activation of the PI3K/AKT signaling pathway in recipient GC cells via ApoE, thus enhancing the migration of GC cells (67).

TAMs are abundant in the TME and can regulate chemotherapy resistance (68). It has been shown that M2 macrophage-derived exosomes containing circTEX2 regulate the miR-145/ABCC1 axis, thereby increasing GC cell cisplatin resistance. These data suggest that exosome transfer between macrophages and cancer cells may be a robust target for reducing cisplatin resistance in GC (69). Furthermore, circ0008253 from M2-polarized TAM-derived exosomes can be transferred from TAMs to GC cells, ultimately enhancing GC cell resistance to oxaliplatin (70). Furthermore, the lncRNA CRNDE is enriched in exosomes derived from M2-polarized TAMs and can be transferred to GC cells. Mechanistically, CRNDE promotes NEDD4-1-mediated PTEN ubiquitination and reduces cisplatin resistance in GC (71). Additional work demonstrated that exosomal miR-588 secreted by M2 macrophages encourages cisplatin resistance in GC cells by partially targeting CYLD (72).

Tumor-associated neutrophils (TANs) play dual roles in tumors, where N1 TANs have anti-tumor functions and N2 TANs exhibit pro-tumor activities (73). Neutrophil-derived exosomes regulate the initiation and progression of tumors by delivering mRNA, miRNA, and piRNA molecules. Studies have shown that exosomes from N2 TANs transfer miR-47445-5p/miR-3911 to GC cells, down-regulating the expression of slit guidance ligand 2 (SLIT2) and promoting GC metastasis (74).

## 4 The role of exosomes in GC immunotherapy

### 4.1 Exosomes can augment GC immunotherapy

Many recent studies have revealed that exosome-mediated crosstalk between cancer cells and immune cells in the TME can impact the outcome of immunotherapy (16) (**Figure 1C**). For example, γδ T cells play crucial roles in innate and adaptive immune surveillance and are receiving increasing attention in the context of cancer immunotherapy. Immunotherapies based on γδ T cells have shown favorable safety profiles and clinical responses in patients with a variety of cancers (75). Indeed, studies have demonstrated that thrombospondin 1 (THBS1) in exosomes derived from GC cells regulates m6A modification in Vγ9Vδ2T cells and activates the retinoic acid-inducible gene-I (RIG-I) receptor signaling pathway, leading to increased Vγ9Vδ2T cell cytotoxicity toward GC cells. Thus, targeting the exosomal THBS1/m6A/RIG-I axis could be of great significance for Vγ9Vδ2T cell-based GC immunotherapy (16). Furthermore, exosomes facilitate the transfer of miR-451 from GC cells to infiltrating T cells, leading to an increase in T cell Th17 polarization via reduced AMP-activated protein kinase (AMPK) and enhanced mammalian target of rapamycin (mTOR) activity (76).

Macrophage-derived exosomes carrying ncRNAs and immune factors can promote immune activation by regulating B cells, T cells, and NK cells (77). Studies have demonstrated that exosomes derived from M1 macrophages, which contain miR-16-5p, can initiate T cell immune responses and impede the formation of GC tumors by decreasing the expression of PD-L1 (78). Moreover, exosomal circ0017252 released from GC cells can efficiently suppress M2-like polarized macrophages and inhibit the invasion and malignant progression of GC cells by sequestering miR-17-5p (79).

### 4.2 Exosomes can be used as delivery vehicles for GC treatment

Although numerous cytotoxic chemotherapeutic agents, targeted therapies, and immunomodulators have demonstrated remarkable cancer treatment efficacy, challenges such as drug resistance and side effects remain, and the development of new approaches is crucial. Recent studies indicate that the loading of therapeutic agents into nanoparticles designed specifically to target GC may improve treatment outcomes and greatly reduce adverse effects (80) (**Figure 1C**). For example, some studies have investigated the effects of a tumor-targeting nanosystem, in combination with chemotherapy and immunotherapy, on GC treatment and prognosis. Specifically, a tumor-targeting system based on a fusion vector of modified iPSC and DC exosomes, DOX@aiPS-DCexo, was developed and modified with an anti-PD-1 antibody. Additionally, when the chemotherapy drug doxorubicin (DOX) was loaded into the DOX@aiPS-DCexo fusion system, it was capable of specifically targeting and eliminating tumor tissues. The system also had the ability to activate and enhance a range of local immune responses and mitigate tumor-associated immunosuppression, highlighting the efficacy of combined chemotherapy and immunotherapy treatment (81). In addition, the efficacy of aggregation-induced emission luminogen (AIEgen)-based photodynamic therapy (PDT) is constrained by cellular glutathione (GSH), the latter of which must be reduced to effectively induce oxidation within tumor cells. Consistent with this observation, studies have leveraged tumor-derived exosomes for the co-delivery of AIEgens and proton pump inhibitors in the context of tumor combination treatment. This system can restrain cell glutamine metabolism, inhibit the generation of GSH and ATP in tumor cells, improve the effect of AIEgen type I PDT, and promote immunogenic tumor death (82). Other studies have described the development of engineered exosomes, consisting of genetically engineered adipose-derived stem cells that express the MKN45-binding peptide DE532 on their surfaces and in which 17-(dimethylaminoethyl amino)-17-demethoxygeldanamycin (17-DMAG) has been encapsulated. These targeted, 17-DMAG-loaded DE532 exosomes effectively delivered anti-cancer agents, enhancing the therapeutic responses of GC (13). Moreover, the lipid carrier protein prostaglandin D2 synthase (L-PGDS) has been shown to inhibit GC growth. L-PGDS-loaded EVs (EVs-L-PGDS) were produced by transducing MSCs with adenovirus encoding L-PGDS. These EVs-L-PGDS decreased the expression of stem cell markers such as Oct4, Nanog, and Sox2, and blocked STAT3 phosphorylation, suppressing GC tumor development and suggesting that MSC-derived EVs can act as efficient nano-capsules (83).

In addition, exosome-mediated delivery of RNA has demonstrated remarkable cancer treatment potential. Indeed, exosome-mediated siRNA, miRNA, and anti-miRNA oligonucleotide delivery has been extensively studied in the treatment of diverse cancers; modification of these exosomes through engineering can further improve their targeting capacity and therapeutic efficacy (84). Studies have demonstrated that circDIDO1 suppresses GC progression via the regulation of the miR-1307-3p/SOSC2 axis and that the use of RGD-modified exosomes with circDIDO1 (RGD-Exo-circDIDO1) can reduce GC occurrence and invasion. These results suggest that engineered RGD-Exo-circDIDO1 could represent a feasible nanomedicine for GC treatment (85). Additionally, exosomes acting as nanoparticles impede tumor advancement and angiogenesis in GC by transporting hepatocyte growth factor (HGF) siRNA (86). In another study, the quantity of miR-29b within the peritoneal exosomes of patients with pre-metastases (PMs) was markedly reduced. Transduction of human bone marrow-derived MSCs with an integrated recombinant lentiviral vector encoding miR-29b confirmed that sEVs from bone marrow MSCs were effective carriers of miR-29 that could inhibit the development of PM in GC (87). Still another study used electroporation to insert miR-13896 into human umbilical cord MSC-EVs. These engineered EVs were effectively transported to tumor sites where miR-13896 specifically targets and down-regulates the ATG2A-mediated autophagy pathway, thereby significantly suppressing the growth and metastasis of GC cells (88). Furthermore, exosomes were utilized to deliver anti-miR-214, with the aim of reversing cisplatin-based chemotherapy resistance in GC, and successfully suppressed tumor growth (89).

## 5 Discussion

GC poses a global healthcare challenge. By 2040, it is projected that the incidence of GC will rise by 62%, resulting in a substantial burden on public health services, costs, and patient quality of life (90). GC patients treated with chemotherapy and surgery have a poor prognosis, and many current clinical trials of late-stage tumors are evaluating targeted agents and immunotherapies (91). The GC TME is a highly structured ecological system containing cancer cells, immune cells, CAFs, endothelial cells, pericytes, and various other cell types. These elements work together to sustain proliferation signals, initiate invasion and metastasis, and suppress immune reactions (92). Exosomes are important messengers between cancer cells and TME cells. Indeed, preliminary studies have shown that exosomes generated by cancer cells control the phenotypes and functions of TME cells, driving tumor growth, metastasis, and the emergence of treatment resistance. Exosomes derived from TME cells also contain a broad spectrum of bioactive molecules and have been implicated in the regulation of tumor malignancy (74).

Exosomes play crucial roles in GC-associated immune responses. Specifically, numerous studies have shown that tumor-derived exosomes affect the differentiation, proliferation, and functional regulation of various immune cell populations in the TME, including T cells, macrophages, neutrophils, and MDSCs (93). This review focuses on the complex mechanisms by which tumor-derived exosomes regulate GC progression, the immune microenvironment, and immune escape. In addition, exosomes derived from immune cells can also regulate immune responses to GC cells and reshape the immune microenvironment through the delivery of biological molecules such as ncRNAs. Therefore, targeting exosome-secreting immune cells may represent a promising approach to improve the efficacy of GC immunotherapy (94). Furthermore, exosome biogenesis is intricately regulated. GC cells regulate exosome secretion to facilitate pre-metastatic niche formation (95). Meanwhile, crosstalk between cancer cells and other cells in TME via signal transduction enhances exosome biogenesis, which in turn modulates the TME (96). Hypoxia, for instance, also promotes GC exosome release through a HIF-1α-dependent pathway, impacting tumor development and metastasis (97). Therefore, the study of exosomes in the immune microenvironment of GC tumors can facilitate the development of more personalized targeted therapy and immunotherapy regimens for GC patients; improve our understanding of the molecular mechanisms underlying GC proliferation, progression, metastasis, and treatment resistance; and reveal new diagnostic and prognostic biomarkers and potential immunotherapeutic targets.

The success of immunotherapy, which aims to reinstate normal anti-tumor immune responses, reinitiate anti-tumor immunity, and further eliminate tumor cells, demonstrates that immune escape is crucial for tumor development and growth (98). Immunotherapy for GC has greatly improved in recent years, but there is still a paucity of targets that reliably evoke anti-tumor immunity, and challenges persist with respect to achieving precise and personalized GC immunotherapy results (99). Due to their innate capacity for long-distance communication, outstanding biocompatibility, and ability to traverse barriers, exosomes are ideal carriers for the delivery of various molecules, including proteins, nucleic acids, chemotherapy drugs, and gene therapy molecules (80), with great potential for GC immunotherapy applications. Moreover, there has been a lot of evidence indicating that cancer immunotherapy can also target the production of exosomes. For example, tumor cells directly inhibit T cell function by releasing PD-L1 through exosomes, while anti-PD-1 therapy promotes cancer treatment by blocking the PD-1/PD-L1 signaling pathway and reducing the secretion of PD-L1 in exosomes (100).

In the present work, we have explored the possibility of augmenting GC immunotherapy with exosome-loaded molecules and proposed the potential of exosomes as therapeutic delivery carriers. Nevertheless, the utilization of exosomes in this context is still impeded by numerous risks and challenges, including the precision and standardization of exosome extraction procedures, the need to enhance the specificity and detection efficacy of techniques such as liquid biopsy, and the absence of clinical sample verification (101). In addition, given their immunomodulatory effects, the use of exosomes as carriers to construct targeted chemotherapy drugs may become a new approach for personalized GC treatment. Accordingly, there is an urgent need to increase the efficiency of loading drugs or antigens into exosomes and develop more convenient methods to evaluate this process.

In summary, the molecular mechanisms underlying exosome-mediated GC occurrence, development, and immune escape or immune activation should be clarified. Moreover, targeted exosomes that amplify anti-tumor immune responses should be explored so as to overcome challenges in the standardization and clinical application of exosomes. Promoting the development of novel exosome-dependent or exosome-targeted drugs will help achieve precise delivery and synergistic therapy in the context of GC immunotherapy.

## 6 Conclusion

In conclusion, exosomes mediate communication between GC cells and other cell types within the TME. Exosomes regulate cancer initiation, progression, metastasis, and immune responses by delivering different biomolecules. Herein, we summarized recent studies on the molecular mechanisms underlying exosome-mediated GC development. We also described the role of exosomes as biomarkers for GC diagnosis and treatment, focusing on how exosomes derived from GC or immune cells modulate GC immune escape. Moreover, exosomes are promising vectors for targeted drug delivery and have great potential in GC immunotherapy applications. More extensive studies are needed to thoroughly understand the regulatory mechanisms by which exosomes are released by cells in the immune GC microenvironment and further explore the utility of exosomes in the augmentation of immunotherapy. Together, this work will facilitate the development of novel diagnostic, prognostic, and therapeutic strategies and targets.
