# Supplementary material for: Recent progress in the study of exosomes in the gastric cancer immune microenvironment
Source: Front Immunol. 2025 Jul 23;16:1595124. doi: 10.3389/fimmu.2025.1595124 (PMC12325315; doi:10.3389/fimmu.2025.1595124)
Supplement: Supplementary file 1 [file DataSheet1.zip › 批注文件/exosome GC 1.0 HaiboLiu.docx]

**Recent research progress of exosomes in the immune microenvironment of gastric cancer**

**Abstract**

Gastric cancer (GC) is one of the most common types of cancer and a leading cause of cancer-related death. There is an urgent need to explore new directions in the treatment of GC to enhance diagnosis, treatment and patient prognosis. Exosomes are nanoscale extracellular vesicles, which originate from a variety of cells and contain DNA, RNA, proteins, lipids and other bioactive components, and play an important role in the development of GC and the regulation of tumor microenvironment (TME). Tumor-derived exosomes can promote tumor cell development and change TME and immune response, while exosomes derived from immune cells can also regulate immune function and regulate tumor immune microenvironment. Immunotherapy is one of the breakthroughs in the treatment of GC, and immunotherapy combined with exosome targeted therapy is expected to be an effective treatment. A large number of studies have shown that exosomes play a key role in the growth, immune escape, immune microenvironment remodeling and immunotherapy of GC. This review describes the role of exosomes in the microenvironment of GC, focuses on the mechanism by which exosomes regulate the immune response of GC, and summarizes the current status and challenges in the development of exosome-based diagnosis and immunotherapy for GC.

**Introduction**

Gastric cancer (GC) is the fourth leading cause of cancer-related death worldwide and the fifth most common disease worldwide [1]. Factors affecting the development of GC include genetic polymorphism, environmental exposure, age, sex and Helicobacter pylori [2]. Due to the low rate of early diagnosis and the lack of specific clinical symptoms, GC is usually diagnosed at an advanced stage of metastasis, with a 5-year survival rate of only about 32% [3]. Surgical resection, perioperative chemotherapy, adjuvant chemotherapy, radiotherapy, immunotherapy, and targeted therapy are effective treatment options for GC, but even after surgery, about 60% of patients experience local recurrence or distant metastasis [4]. Therefore, it is important to explore new and effective biomarkers and therapeutic strategies to improve the prognosis and quality of life of patients with GC. The prognosis and response to immunotherapy in patients with GC are also largely influenced by the morphological and molecular heterogeneity of the tumor microenvironment (TME). Immunomodulatory cells in GC TME include regulatory T cells (Treg), tumor-infiltrating medullary suppressor cells (MDSC), tumor-associated macrophages (TAM) and natural killer cells (NK cells) [5]. Immune cells and cancer cells can interact to influence the onset and progression of cancer.

Exosomes are a subpopulation of extracellular vesicles (EVs) with a diameter of 30-150 nm that are secreted by virtually all types of cells and can be stably present in a variety of biological fluids [6]. Exosomes are composed of lipid bilayer containing transmembrane proteins and contain cytoplasmic proteins, lipids and nucleic acids, capable of mediating local and distant cell communication by transferring specific cargo under physiological and pathological conditions [7]. In the TME, exosome transport of signaling molecules that drive many biological processes has been shown to have diagnostic and therapeutic functions in several studies [8]. Many studies have shown that exosome-delivered contents play a crucial role in the proliferation, metastasis, drug resistance, immune response, angiogenesis and treatment of GC [9]. The use of exosomes in the field of cancer has received a lot of attention, and because exosomes are more stable than proteins and hormones in the blood, studies have shown that exosomes can be used as early biomarkers for cancer detection and disease progression [10]. In addition, advances in exosome engineering technology have led to the use of targeted exosomes as a promising avenue to deliver therapeutic drugs focused on cancer treatment [11]. With their biocompatibility, low immunogenicity, and ability to transport biomolecules between cells, exosomes can also cross biological barriers and are suitable for the targeted delivery of various therapeutic agents (such as small molecules, siRNA, and miRNA) to specific cellular targets in organisms [12].

The occurrence and development of GC is closely related to immune cells and other types of mesenchymal stromal cells (MSCs), cytokines and exosomes in TME [13]. Exosomes derived from tumor cells or related immune cells can carry immunosuppressive factors that regulate immune cell activity and help tumors evade immune surveillance [14]. It is of great significance to explore how exosomes regulate the immune microenvironment of GC through the transmission of signaling molecules. In this review, we summarize the mechanism of exosomes regulating the development of cancer in GC, and focus on the recent studies on exosome-mediated immune escape in GC. In addition, tumor immunotherapy, including immune checkpoint inhibitors, cellular immunotherapy and therapeutic cancer vaccines, has aroused great interest in GC research and clinical practice. Identifying key targets and characterizing molecular mechanisms involved in GC immunity will help reveal the pathogenesis of GC and enhance the efficacy of immunotherapy [15]. The application of exosomes to promote immune response in GC and the targeted treatment of exosomes as drug carriers were reviewed, which provided a basis for potential strategies for the treatment of GC targeting exosomes and immune microenvironment.

**1. The role of exosomes in gastric cancer**

A large amount of evidence shows that exosomes are closely related to the progression of gastric cancer (GC) through the delivery of functional biomolecules, including tumorigenesis, metastasis, angiogenesis, immune escape and drug resistance [16]. The occurrence and development of cancer is often influenced by the interaction of tumor cells or the microenvironment, in which bioactive substances carried by exosomes are involved in many important processes, including exosomal proteins, miRNAs, lncRNAs, and circRNAs [17].

Recent studies have shown that the exosomal circ_0001789 regulates the malignant phenotype of GC cells, and the circ_0001789 sponge miR-140-3p regulates PAK2 to promote the progression of GC [18]. Besides, the researchers found that the exosomal LINC00853 from GC cells promotes epithelial-mesenchymal transformation through the MAP17/PDZK1/AKT signaling pathway, which in turn promotes the progression of GC [19]. Other studies have found that tumor-derived exosomal LINC01480 can up-regulate the expression of VCAM1 through competitive binding with miR-204-5p, thereby promoting the proliferation, migration, invasion and EMT of GC cells, suggesting that LINC01480 may be a potential therapeutic target for GC [20]. Another study showed that the exosomal circSHKBP1 sponges miR-582-3p to increase HUR expression and enhance the stability of VEGF mRNA. And circSHKBP1 directly binds to HSP90 and blocks the interaction between STUB1 and HSP90, inhibits the ubiquitination of HSP90 and promotes the progression of GC [21]. In addition, exosomal circRELL1 can inhibit the malignant development of GC by regulating autophagy activation in GC, resulting in the spongification of miR-637 and indirectly regulating the expression of EPHB3 [22]. Other studies have shown that exosomes containing miR-15b-3p from GC can enhance tumorigenesis and malignant transformation in GC through the DYNLT1/Caspase-3/Caspase-9 signaling pathway [23]. Moreover, exosomes transfer LINC01559 activates the PI3K/AKT pathway and accelerates GC progression by up-regulating PGK1 and down-regulating PTEN [24]. Additionally, it has been found that exosome-delivered circSTAU2 may act as a tumor suppressor to inhibit the progression of GC through the miR-589/CAPZA1 axis [25].

Exosomes can mediate GC transfer to local or distant tissues and organs. More than half of GC patients are found to have lymph node metastases at initial diagnosis, often leading to tumor metastasis to other organs and a poor prognosis for GC [26]. Exosomal lncAKR1C2 secreted by GC cells was shown to encode pep-AKR1C2 in lymphatic endothelial cells and promote CPT1A expression by regulating YAP phosphorylation, leading to enhanced fatty acid oxidation and ATP production. It can also enhance the tube formation and migration of lymphatic endothelial cells, and promote lymphangiogenesis and lymphatic metastasis *in vivo* [27]. The formation of premetastatic niches (PMN) in distant organs plays a crucial role in tumor metastasis [28]. Studies have shown that when human umbilical vein endothelial cells ingested exosomal NOS3 derived from GC cells, they showed increased nitric oxide levels, induced angiogenesis, established liver PMN, and ultimately promoted the occurrence of liver metastasis of GC [29]. Furthermore, exosomes derived from GC cells promote the growth and metastasis of GC by transporting FRLnc1, suggesting that FRLnc1 in exosomes may be a potential biomarker for the diagnosis and treatment of GC [30]. Other studies have found that exosomes from peritoneal lavage in GC contain a large number of miRNAs associated with peritoneal metastasis. Among them, has-let-7g-3p and hsa-miR-10395-3p can be used as biomarkers to predict peritoneal metastasis and chemotherapy efficacy, and participate in GC metastasis [31].

Exosomes also mediate intercellular communication during cancer progression and promote therapeutic resistance. At present, cisplatin based chemotherapy has become the main treatment option for patients with advanced GC, but many patients develop resistance to cisplatin due to epigenetic modifications, signaling pathway changes, and cell metabolism disorders [32]. CircHIPK3 has been found to lead to cisplatin resistance in GC by blocking autophagy dependent iron death, and the serum exosomal circHIPK3 may be a noninvasive indicator to evaluate cisplatin resistance in GC [33]. In addition, miR-769-5p delivered by exosomes from cisplatin-resistant GC cell lines confers cisplatin resistance to GC and promotes cancer progression by targeting CASP9 and promoting ubiquitination degradation of p53 [34]. Linc00852 of exosomes derived from cisplatin-resistant GC cells also regulates COMMD7 through miRNA-514a-5p to promote cisplatin resistance in recipient cells [35]. Cancer-associated fibroblasts (CAFs) are the dominant stromal cell types in the tumor microenvironment [36]. It was found that CAFs secreted exosomal miR-522 inhibits iron death in cancer cells by targeting ALOX15 and blocking the accumulation of lipid-ROS, mediating acquired chemotherapy resistance in GC [37].

Early detection and diagnosis of GC is crucial to improve the prognosis of patients. Liquid biopsy is a non-invasive method for detecting circulating tumor cells, circulating tumor DNA and extracellular vesicles in serum and other body fluids [38]. Exosomal non-coding RNA (ncRNA) has shown great application prospects as a molecular biomarker for cancer diagnosis [39]. Studies have identified 182 candidate GC biomarkers in serum exosomes by RNA sequencing, and constructed exosomal ncRNA characteristics through machine learning for non-invasive and early detection in patients with GC, providing a promising liquid biopsy method for enhancing the early diagnosis of GC [40]. Besides, liquid biopsy characteristics of circulating exosome-derived mRNA, miRNA, and lncRNAs can predict the therapeutic effect of neoadjuvant chemotherapy (neoCT) in patients with advanced GC [41]. Other studies have shown that circulating EVs-derived lncRNA-GC1 is an early marker of the efficacy of neoCT and can predict the survival rate of GC patients treated with neoCT [42]. Additionally, exosomal PD-L1 is associated with systemic inflammatory markers, immunomodulatory cytokines, and T cells, and serum-derived exosomal PD-L1 may reflect immunosuppressive status in patients with advanced GC [43]. Exosomal lncRNA HOTTIP has also been identified as a potential novel diagnostic and prognostic biomarker for GC [44].

**Table 1**

**2. Exosomes can mediate immune escape of gastric cancer**

**2.1 Exosomes from gastric cancer cells mediate immune escape in gastric cancer**

Tumor cells can regulate the immune environment through the release of exosomes, and many studies have shown that exosomes contribute significantly to the reprogramming of the tumor microenvironment, which helps cancer cells evade the immune system [45]. Therefore, the exploration of exosom-related molecular classification is of great significance to reveal different immune escape mechanisms in GC [46].

T cell activation is central to the anti-tumor immune response [47]. Some studies have found that exosomal circMAN1A2 can promote the progression of GC *in vitro and in vivo*, and inhibit the antitumor activity of T cells. CircMAN1A2 competes with FBXW11 to bind to SFPQ, stabilize SFPQ expression and inhibit the activation of T cell receptor signaling pathways in T cells, thereby reducing its antitumor activity [48]. Other studies have shown that exosomes isolated from GC cell lines can change the gene expression and cytokine secretion levels of CD8+T cells, increase the frequency of effector memory CD4+T and MDSC, and reduce the frequency of CD8+T cells and NK. Mice injected with GC cell exosomes develop immunosuppressive TME in the lungs. It was demonstrated that exosomes from GC cells regulate the immunosuppressive TME by modulating immune function [49]. Moreover, studies have found that LSD1 inhibits the response of T cells in the GC microenvironment by inducing the accumulation of PD-L1 in GC exosomes, providing a new target for immunotherapy against GC [50]. It was also found that exosomes mediated the redistribution of miRNA-451 from cancer cells to infiltrating T cells and increased Th17 polarization of these T cells through decreased AMPK and increased mTOR activity [51]. Furthermore, studies have shown that GC cell-derived exosomes can be effectively taken up by Vγ9Vδ2 T cells, and exosomal miR-135b-5p is delivered to Vγ9Vδ2 T cells. It impairs the function of Vγ9Vδ2 T cells by targeting specific protein 1 (SP1), induces apoptosis, and reduces the production of cytotoxic cytokines IFN-γ and TNF-α. Targeting exosomal miR-135b-5p/SP1 axis may improve the efficiency of Vγ9Vδ2 T cell-based GC immunotherapy [52].

Tumor-associated macrophages (TAMs), especially M2-polarized TAMs, can be recruited and driven by tumor-derived inflammatory cytokines and immunosuppressive metabolites, and are important factors in GC tumor progression, immune escape, and therapeutic resistance [53]. Studies have shown that in GC cells, elevated SERPINE1 expression increases the level of let-7g-5p in exosomes, thereby promoting the transfer of let-7g-5p to macrophages through cancer-derived exosomes. The exosomal let-7g-5p is internalized by macrophages, down-regulating SOCS7 protein levels, disrupting its interaction with STAT3 and unlocking inhibition of STAT3 phosphorylation, resulting in over-activation of STAT3, which drives M2 polarization [54]. Liver metastasis (LM) is a major obstacle to the prognosis of patients with GC. GC-derived exosomes mainly accumulate in the liver and are internalized by intrahepatic macrophages, and the expression level of miR-519a-3p in serum exosomes of GC-LM patients is significantly higher than that of patients without LM. Exosomal miR-519a-3p activates the MAPK/ERK pathway by targeting DUSP2, resulting in M2-like polarization of macrophages. M2-like polarized macrophages accelerate GC-LM by inducing angiogenesis and promoting intrahepatic pre-metastatic niche formation [55]. In addition, other studies have shown that PLXNC1 inhibits the SOCS7-STAT3 interaction by transferring the exosomal miR-92b-5p derived from GC cells, and subsequently activates STAT3 in macrophages, promoting the proliferation of GC cells and the M2 polarization of TAMs [56]. It has also been found that exosomal circGLIS3 can promote GC metastasis and macrophage M2 type polarization. Mechanistically, circGLIS3 can sponge miR-1343-3p, up-regulate PGK1 expression, and regulate VIMENTIN phosphorylation to promote GC tumorigenesis [57]. Additionally, ELNF1-AS1 is highly enriched in GC derived exosomes, which target and regulate miR-4644, thereby triggering PKM expression. Exosomal ELNF1-AS1 can also regulate glycolysis through PKM in HIF-1α-dependent manner in GC, promote M2 polarization and macrophage recruitment, and thus enhance the growth and metastasis of GC cells [58]. Another study demonstrated that GC cells can also induce macrophage M2 polarization through the DUSP3/JAK2/STAT3 pathway mediated by exosomal miR-541-5p [59]. Moreover, HMGB1 in GC cell-derived exosomes interacts with the transcription factor POU2F1 to inhibit the transcriptional activity of p50, resulting in the inactivation of the NF-κB signaling pathway, thereby inducing the polarization of M2-like macrophages [60].

Melatonin (MLT) is a hormone with potential anti-tumor properties. MLT can regulate the relevant miRNA in cancer-derived exosomes to regulate the level of PD-L1 in macrophages and increase the secretion of TNF-α and CXCL10 by macrophages, thereby inhibiting tumor growth. The experimental results demonstrate that MLT regulates the tumor immune microenvironment by regulating exosomes derived from GC cells [61]. Other studies have shown that GC derived exosomes can effectively induce the production of PD1-expressing tumor-associated macrophages TAMs, and these cells can produce a large amount of IL-10, which damages the function of CD8+T cells, thus creating conditions to promote the progression of GC [62].

Myeloid suppressor cells (MDSC) are the main immunosuppressive cells in TME, and the increase of PD-L1 expression in gastric epithelium can increase the number of MDSC in infiltrating tumor [63]. Studies have shown that exosomal PD-L1 derived from GC may induce immunosuppression by promoting the aggregation of MDSCs, and exosomal PD-L1 may stimulate MDSC proliferation by triggering the IL-6/STAT3 signaling pathway *in vitro* [64]. Neutrophils are also important players in cancer development and progression and have been shown to promote carcinogenesis, growth and metastasis, angiogenesis and immunosuppression [65]. Some studies have shown that neutrophils can promote tumor phenotypes through tumor polarization. It has been demonstrated that exosomes derived from GC cells induce autophagy of neutrophils and promote tumor activation through HMGB1/TLR4/NF-κB signaling [66]. In addition, EVs from the GC microenvironment transported HMGB1 to activate STAT3 and upregulated PD-L1 gene expression in neutrophils to inhibit T cell immunity, illustrating the multifaceted role of EVs in coordinating the immunosuppressive microenvironment [67]. Natural killer (NK) cells play a key role in maintaining immune homeostasis and inhibiting tumor formation, and reduced NK cell efficacy has been observed in GC tissue and peripheral blood [68]. Studies have shown that the exosomal miR-552-5p from GC cells can promote the progression of GC by regulating the PD-1/PD-L1 axis to affect NK cell function and subsequently affect the epithelial-mesenchymal transformation (EMT) of GC [69].

**2.2 Exosomes from immune cells mediate immune escape in gastric cancer**

A large amount of evidence shows that exosomes secreted by immune cells can also be transmitted in the TME and play an important role in regulating tumor progression. The multifunctional role of M2 TAMs exosomes in cancer progression has been extensively studied. It has been found that the M2 TAMs exosomal MALAT1 interacts with δ-catenin protein and inhibits its ubiquitination and degradation through β-TRCP. And MALAT1 upregulates HIF-1α expression by acting as a sponge for miR-217-5p, leading to enhanced aerobic glycolysis in GC cells. These results suggest that M2-TAM-derived exosomes promote GC progression through MALAT1-mediated glycolytic regulation, providing a potential target for GC therapy [70]. Besides, TAMs are a unique group of immune cells that express ApoE in the GC microenvironment. Studies have shown that M2 TAM-derived exosomes mediate the intercellular transfer of ApoE-activated PI3K-Akt signaling pathway in the receptor GC cells, thereby promoting the migration of GC cells [71].

TAMs are abundant in the TME and can regulate chemotherapy resistance in tumor cells [72]. The exosomal circTEX2 of M2 macrophage has been found to enhance cisplatin resistance in GC via miR-145/ABCC1, suggesting that communication between macrophages and cancer cells via exosomes may be a promising therapeutic target for the treatment of cisplatin resistant GC [73]. Furthermore, circ0008253 contained in M2-polarized TAMs derived exosomes can be transferred from TAMs to GC cells, ultimately enhancing oxaliplatin resistance in GC cells [74]. Another study found that lncRNA CRNDE is enriched in exosomes derived from M2-polarized TAMs and transferred from M2 TAMs to GC cells. Mechanistically, CRNDE promotes NEDD4-1 mediated PTEN ubiquitination and reduces cisplatin resistance in GC [75]. The researchers also found that the exosomal miR-588 from M2 macrophages promotes cisplatin resistance in GC cells by partially targeting CYLD [76].

Tumor-associated neutrophils (TANs) play a dual role in tumors, where N1 TANs has anti-tumor function and N2 TANs has pro-tumor activity [77]. Neutrophil-derived exosomes participate in the genesis and progression of tumors by delivering mRNA, miRNA and piRNA. Studies have shown that exosomes from N2 TANs transfer miR-47445-5p /3911 to GC cells to down-regulate the expression of SLIT2, thus promoting the metastasis of GC [78].

**3. The role of exosomes in immunotherapy of gastric cancer**

**3.1 Exosomes can promote immunotherapy of gastric cancer**

Recently, many studies have reported that the ability of exosomes to mediate communication between tumor cells and various immune cells in the TME can affect the outcome of immunotherapy [15]. γδ T cells are an important subtype of T cells, which play a crucial role in innate and adaptive immune surveillance, and are receiving increasing attention in the application of cancer immunotherapy. Immunotherapies based on γδ T cells have shown a good safety profile and clinical response in patients with a variety of cancers [79]. It has been found that the exosomal THBS1 derived from GC cells significantly enhances the cytotoxicity of Vγ9Vδ2 T cells to GC cells and the production of IFN-γ, TNF-α, perforin and granase B. The exosomal THBS1 can regulate METTL3 or IGF2BP2-mediated m6A modification to further activate the RIG-I like receptor signaling pathway in Vγ9Vδ2T cells, thereby enhancing the function of Vγ9Vδ2T cells. Targeting the exosomal THBS1/m6A/RIG-I axis may have important implications for the immunotherapy of GC based on Vγ9Vδ2 T cells [15].

Macrophage-derived exosomes carrying ncRNAs and immune factors can generate immune activation by regulating B cells, T cells, and NK cells [80]. Studies have shown that M1 macrophage-derived exosomes containing miR-16-5p can trigger T cell immune responses and inhibit GC tumor formation *in vitro* and *in vivo* by reducing the expression of PD-L1 [81]. Besides, the exosomal hsa_circ_0017252 secreted by GC cells can effectively inhibit M2-like polarization of macrophages, and then inhibit the invasion and malignant progression of GC cells through sponge miR-17-5p [82].

**3.2 Exosomes can be used as delivery carriers for the treatment of gastric cancer**

Although many cytotoxic chemotherapeutic agents and targeting and immunomodulators have shown strong performance in cancer treatment, there are still problems such as drug resistance and side effects, and new approaches to cancer treatment are crucial. Existing studies have shown that encapsulating therapeutic drugs in nanoparticles specifically targeted for GC is expected to improve the therapeutic efficacy of GC and significantly reduce adverse effects [83]. There have been studies on the application of tumor targeting nanosystem combined with chemotherapy and immunotherapy in the treatment and prognosis of GC. A tumor targeting system based on modified iPSC-exos and DC-exos fusion vectors DOX@aiPS-DCexo was developed, and the fusion vector was modified with PD-1 antibody. To alleviate the immunosuppression of lymphocytes in tumor tissues and enhance the efficacy of immunotherapy, after packaging chemotherapy drug doxorubicin (DOX), DOX@aiPS-DCexo fusion system can target and destroy tumor tissues, awaken and amplify a series of anti-tumor immune responses, and realize the synergistic treatment of chemotherapy and immunotherapy [84]. In addition, the efficiency of aggregation-induced emission luminogen (AIEgens) -based photodynamic therapy (PDT) is limited by cellular glutathione (GSH), which needs to be reduced to effectively induce oxidation within tumor cells. Recent studies have shown that tumor cell metabolism is primarily dependent on glutamine, which is also a source of nitrogen and ATP for GSH synthesis. Therefore, glutamine-based starvation therapy may effectively enhance photodynamic therapy. Tumor-derived exosomes are used for co-delivery of AIEgens and proton pump inhibitors (PPI) for tumor combination therapy. PPI inhibits cell glutamine metabolism, inhibits the production of GSH and ATP in tumor cells, and improves the effect of AIEgens type I PDT, thereby promoting tumor immunogenic death [85]. Studies have also been conducted by genetically engineering adipose-derived stem cells to express the MKN45-binding peptide (DE532) on their surfaces, and encapsulating 17- (dimethylaminoethyl amino) -17-demethoxygel danamycin (17-DMAG) in engineered exosomes. DE532 exosomes loaded with 17-DMAG were obtained, and targeted exosomes delivered anticancer agents to effectively enhance the therapeutic effect of GC [11]. Moreover, the lipid carrier protein prostaglandin D2 synthase (L-PGDS) has been shown to inhibit the growth of GC. EVs-L-PGDS was produced by MSCs transfected with adenovirus encoding L-PGDS. EVs-L-PGDS decreased the expression of stem cell markers, including Oct4, Nanog, and Sox2, and inhibited STAT3 phosphorylation in GC cells, significantly inhibiting GC tumor progression, suggesting that MSC-derived EVs could be used as effective nano-capsules [86]. Another study isolated exosomes after transfection of HEK293T cells with si-c-Met, and found that exo-si-c-Met could inhibit the invasion and migration of GC cells, promote cell apoptosis, and reverse the resistance of GC to cisplatin [87].

Notably, exosome-based RNA delivery has also shown great promise in the field of cancer therapy. Exosome-mediated siRNA, miRNA or anti-miRNA oligonucleotide delivery has been widely studied in the treatment of various cancers, and the engineering modification of exosomes has further improved the targeting ability and therapeutic effect of exosomes [88]. Studies have shown that circDIDO1 inhibits the progression of GC by regulating the miR-1307-3p/SOSC2 axis, and the application of RGD-modified circDIDO1-supported exosomes can inhibit tumorigenicity and invasiveness of GC. The results indicated that the engineered RGD-Exo-circDIDO1 could be used as a feasible nano-medicine for the treatment of GC [89]. Additionally, exosomes, as nanoparticles, inhibit tumor growth and angiogenesis in GC by delivering hepatocyte growth factor siRNA [90]. The level of miR-29b in the peritoneal exosomes of patients with PM was significantly reduced. Transfection of human bone marrow derived MSCs with an integrated recombinant lentiviral vector of miR-29b proved that sEV derived from bone marrow MSCs was an effective carrier for the administration of miR-29b, which could inhibit the development of PM in GC [91]. Another study applied electroporation to insert miR-13896 into hucMSC-EVs. The engineered EVs can be delivered to tumor sites, and miR-13896 targets and down-regulates the ATG2A-mediated autophagy pathway, significantly inhibiting the growth and metastasis of GC cells [92]. Furthermore, other studies have used exosomes as nanoparticles to deliver anti-miR-214 to reverse chemotherapy resistance to cisplatin in GC, and the treatment of exo-anti-214 *in vivo* and *in vitro* reversed chemotherapy resistance and inhibited tumor growth [93].

**Discussion**

Gastric cancer (GC) is a global healthcare challenge and it is estimated that the incidence of GC could increase by 62% by 2040, which will have a significant impact on public health services, costs and quality of life for patients [94]. For the treatment of GC, patients with chemotherapy and surgical regimens have a poor prognosis, and many current clinical trials use targeted agents and immunotherapies in late stages [95]. The tumor microenvironment (TME) of GC is a highly structured ecosystem of cancer cells, immune cells, cancer-associated fibroblasts (CAFs), endothelial cells (ECs), pericytes, and other cell types involved in maintaining proliferation signals, activating invasion and metastasis, and suppressing immune responses [96]. Exosomes are important messengers between tumor cells and TME cells. Early studies have shown that exosomes derived from tumor cells regulate the phenotype and function of TME cells, thus promoting tumor growth, metastasis and treatment resistance. Exosomes derived from TME cells also contain a variety of bioactive molecules and participate in the regulation of tumor malignancy [78].

Exosomes play an important role in the immune response of GC. Numerous studies have shown that tumor-derived exosomes affect the differentiation, proliferation and functional regulation of various immune cell populations in the TME, including CD8+ T cells, CD4+ T cells, γδ T cells, macrophages, neutrophils, and MDSC [97]. This review focuses on the complex mechanism of tumor-derived exosomes regulating GC progression and immune microenvironment and mediating immune escape of GC. In addition, exosomes derived from immune cells can also regulate the immune response of GC cells and reshape the immune microenvironment through the delivery of biological molecules such as non-coding RNA (ncRNAs). Targeting exosome-secreting immune cells may provide a promising approach to improve the therapeutic efficacy of GC immunotherapy [98]. The study of exosomes in the immune microenvironment of GC tumors can provide more personalized targeted therapy and immunotherapy for GC patients, and better understand the molecular mechanisms of GC proliferation, progression, metastasis, and treatment resistance, revealing new diagnostic/prognostic biomarkers and potential therapeutic targets.

Immunotherapy is a treatment to restore the normal anti-tumor immune response, restart tumor immunity, and further eliminate tumor cells, suggesting that immune escape plays a key role in tumor development and growth [99]. Immunotherapy for GC has made great progress in recent years, but there is still a lack of targets that reliably evoke anti-tumor immunity, and challenges remain in achieving precise and individual immunotherapy for GC and in exploring new targets [100]. Due to their inherent ability to communicate over long distances, excellent biocompatibility, and ability to cross barriers (e.g. cell membranes, blood-brain barrier), exosomes are ideal carriers for the delivery of various molecules, including chemicals, proteins, nucleic acids, and gene therapy agents [83], with great potential for application in GC immunotherapy.

In the present study, the possibility of promoting the immunotherapy of GC by molecules in exosomes has been explored, and a therapeutic method using exosomes as delivery carriers has been proposed. However, the application of exosomes still faces many risks and challenges, such as the accuracy and standardization of exosome extraction methods, the need to improve the specificity and detection efficiency of techniques such as liquid biopsy, and the lack of clinical sample validation, which hinder the wider application of exosomes in GC immunotherapy [101]. In addition, in view of their immunomodulatory effects, the use of exosomes as carriers to construct targeted chemotherapy drugs may become a new approach for personalized treatment of GC. There is an urgent need to improve the efficiency of loading drugs or antigens into exosomes and to develop more convenient methods to evaluate the load efficiency. In summary, the molecular mechanism of exosomes mediating the occurrence and development of GC as well as immune escape or immune activation should be clarified, and exosomes should be targeted to explore therapeutic methods to amplify the anti-tumor immune response, so as to overcome the difficulties in the standardization and clinical application of exosomes. It is helpful to promote the development of future exosome-dependent drugs or exosome-targeted drugs to achieve precise delivery and synergistic therapy, making it one of the potential strategies for GC immunotherapy.

**Conclusion**

In conclusion, exosomes play a role in communication between gastric cancer (GC) cells and their receptor cells in the tumor microenvironment of GC. Exosomes contribute to the regulation of cancer occurrence, progression, metastasis and immune response by delivering different biomolecules. This review specifically summarizes recent studies on the molecular mechanism of exosomes regulating the development of GC and the role of exosomes as biomarkers for the diagnosis and treatment of GC, focusing on the role of exosomes derived from GC cells or immune cells in immune escape of GC. Besides, exosomes are promising vectors for targeted drug delivery and have great potential for application in GC immunotherapy. Further studies are needed to fully understand the regulatory mechanisms of exosomes secreted by cells in the immune microenvironment of GC, and to further address the application of exosomes in immunotherapy to develop their potential for novel targets for diagnosis and prognosis as well as therapeutic strategies.

**Reference**

1. Guan WL, He Y and Xu RH (2023) Gastric cancer treatment: recent progress and future perspectives. J Hematol Oncol 16:57. doi: 10.1186/s13045-023-01451-3

2. Ajani JA, D'Amico TA, Bentrem DJ, Chao J, Cooke D, Corvera C, Das P, Enzinger PC, Enzler T, Fanta P, Farjah F, Gerdes H, Gibson MK, Hochwald S, Hofstetter WL, Ilson DH, Keswani RN, Kim S, Kleinberg LR, Klempner SJ, Lacy J, Ly QP, Matkowskyj KA, McNamara M, Mulcahy MF, Outlaw D, Park H, Perry KA, Pimiento J, Poultsides GA, Reznik S, Roses RE, Strong VE, Su S, Wang HL, Wiesner G, Willett CG, Yakoub D, Yoon H, McMillian N and Pluchino LA (2022) Gastric Cancer, Version 2.2022, NCCN Clinical Practice Guidelines in Oncology. J Natl Compr Canc Netw 20:167-192. doi: 10.6004/jnccn.2022.0008

3. Ferlay J, Colombet M, Soerjomataram I, Mathers C, Parkin DM, Pineros M, Znaor A and Bray F (2019) Estimating the global cancer incidence and mortality in 2018: GLOBOCAN sources and methods. Int J Cancer 144:1941-1953. doi: 10.1002/ijc.31937

4. Ajani JA (2005) Evolving chemotherapy for advanced gastric cancer. Oncologist 10 Suppl 3:49-58. doi: 10.1634/theoncologist.10-90003-49

5. Zhang B, Wang CM, Wu HX, Wang F, Chai YY, Hu Y, Wang BJ, Yu Z, Xia RH, Xu RH and Cao XT (2023) MFSD2A potentiates gastric cancer response to anti-PD-1 immunotherapy by reprogramming the tumor microenvironment to activate T cell response. Cancer Commun (Lond) 43:1097-1116. doi: 10.1002/cac2.12476

6. Kalra H, Drummen GP and Mathivanan S (2016) Focus on Extracellular Vesicles: Introducing the Next Small Big Thing. Int J Mol Sci 17:170. doi: 10.3390/ijms17020170

7. Tkach M and Thery C (2016) Communication by Extracellular Vesicles: Where We Are and Where We Need to Go. Cell 164:1226-1232. doi: 10.1016/j.cell.2016.01.043

8. Miron RJ, Estrin NE, Sculean A and Zhang Y (2024) Understanding exosomes: Part 2-Emerging leaders in regenerative medicine. Periodontol 2000 94:257-414. doi: 10.1111/prd.12561

9. Wang J, Zhang H, Li J, Ni X, Yan W, Chen Y and Shi T (2024) Exosome-derived proteins in gastric cancer progression, drug resistance, and immune response. Cell Mol Biol Lett 29:157. doi: 10.1186/s11658-024-00676-5

10. Jalalian SH, Ramezani M, Jalalian SA, Abnous K and Taghdisi SM (2019) Exosomes, new biomarkers in early cancer detection. Anal Biochem 571:1-13. doi: 10.1016/j.ab.2019.02.013

11. Park JH, Kim SJ, Kim OH and Kim DJ (2024) Enhanced Efficacy of Gastric Cancer Treatment through Targeted Exosome Delivery of 17-DMAG Anticancer Agent. Int J Mol Sci 25. doi: 10.3390/ijms25168762

12. Zhang Y, Li J, Gao W and Xie N (2022) Exosomes as Anticancer Drug Delivery Vehicles: Prospects and Challenges. Front Biosci (Landmark Ed) 27:293. doi: 10.31083/j.fbl2710293

13. Yu X, Zhang Y, Luo F, Zhou Q and Zhu L (2024) The role of microRNAs in the gastric cancer tumor microenvironment. Mol Cancer 23:170. doi: 10.1186/s12943-024-02084-x

14. Kalluri R (2024) The biology and function of extracellular vesicles in immune response and immunity. Immunity 57:1752-1768. doi: 10.1016/j.immuni.2024.07.009

15. Li J, Feng H, Zhu J, Yang K, Zhang G, Gu Y, Shi T and Chen W (2023) Gastric cancer derived exosomal THBS1 enhanced Vgamma9Vdelta2 T-cell function through activating RIG-I-like receptor signaling pathway in a N6-methyladenosine methylation dependent manner. Cancer Lett 576:216410. doi: 10.1016/j.canlet.2023.216410

16. Fu M, Gu J, Jiang P, Qian H, Xu W and Zhang X (2019) Exosomes in gastric cancer: roles, mechanisms, and applications. Mol Cancer 18:41. doi: 10.1186/s12943-019-1001-7

17. Wu H, Fu M, Liu J, Chong W, Fang Z, Du F, Liu Y, Shang L and Li L (2021) The role and application of small extracellular vesicles in gastric cancer. Mol Cancer 20:71. doi: 10.1186/s12943-021-01365-z

18. You J, Chen Y, Chen D, Li Y, Wang T, Zhu J, Hong Q and Li Q (2023) Circular RNA 0001789 sponges miR-140-3p and regulates PAK2 to promote the progression of gastric cancer. J Transl Med 21:83. doi: 10.1186/s12967-022-03853-2

19. Yoon JH, Byun HJ, Kim SY, Jung DH and Lee SK (2024) Exosomal LINC00853 promotes progression of gastric cancer via the MAP17/PDZK1/AKT signaling pathway. Noncoding RNA Res 9:876-886. doi: 10.1016/j.ncrna.2024.03.011

20. Zhang Y, Guo S, Mao T, Guo J, Zhang Q, Tian Z and Li X (2024) Tumor-Derived Exosomal LINC01480 Upregulates VCAM1 Expression by Acting as a Competitive Endogenous RNA of miR-204-5p to Promote Gastric Cancer Progression. ACS Biomater Sci Eng 10:550-562. doi: 10.1021/acsbiomaterials.3c00394

21. Xie M, Yu T, Jing X, Ma L, Fan Y, Yang F, Ma P, Jiang H, Wu X, Shu Y and Xu T (2020) Exosomal circSHKBP1 promotes gastric cancer progression via regulating the miR-582-3p/HUR/VEGF axis and suppressing HSP90 degradation. Mol Cancer 19:112. doi: 10.1186/s12943-020-01208-3

22. Sang H, Zhang W, Peng L, Wei S, Zhu X, Huang K, Yang J, Chen M, Dang Y and Zhang G (2022) Exosomal circRELL1 serves as a miR-637 sponge to modulate gastric cancer progression via regulating autophagy activation. Cell Death Dis 13:56. doi: 10.1038/s41419-021-04364-6

23. Wei S, Peng L, Yang J, Sang H, Jin D, Li X, Chen M, Zhang W, Dang Y and Zhang G (2020) Exosomal transfer of miR-15b-3p enhances tumorigenesis and malignant transformation through the DYNLT1/Caspase-3/Caspase-9 signaling pathway in gastric cancer. J Exp Clin Cancer Res 39:32. doi: 10.1186/s13046-019-1511-6

24. Wang L, Bo X, Yi X, Xiao X, Zheng Q, Ma L and Li B (2020) Exosome-transferred LINC01559 promotes the progression of gastric cancer via PI3K/AKT signaling pathway. Cell Death Dis 11:723. doi: 10.1038/s41419-020-02810-5

25. Zhang C, Wei G, Zhu X, Chen X, Ma X, Hu P, Liu W, Yang W, Ruan T, Zhang W, Wu C and Tao K (2023) Exosome-Delivered circSTAU2 Inhibits the Progression of Gastric Cancer by Targeting the miR-589/CAPZA1 Axis. Int J Nanomedicine 18:127-142. doi: 10.2147/IJN.S391872

26. Kim DH, Choi MG, Noh JH, Sohn TS, Bae JM and Kim S (2015) Clinical significance of skip lymph node metastasis in gastric cancer patients. Eur J Surg Oncol 41:339-45. doi: 10.1016/j.ejso.2014.09.009

27. Zhu KG, Yang J, Zhu Y, Zhu Q, Pan W, Deng S, He Y, Zuo D, Wang P, Han Y and Zhang HY (2023) The microprotein encoded by exosomal lncAKR1C2 promotes gastric cancer lymph node metastasis by regulating fatty acid metabolism. Cell Death Dis 14:708. doi: 10.1038/s41419-023-06220-1

28. Fabris L, Sato K, Alpini G and Strazzabosco M (2021) The Tumor Microenvironment in Cholangiocarcinoma Progression. Hepatology 73 Suppl 1:75-85. doi: 10.1002/hep.31410

29. Hu Y, Zang W, Feng Y, Mao Q, Chen J, Zhu Y and Xue W (2024) mir-605-3p prevents liver premetastatic niche formation by inhibiting angiogenesis via decreasing exosomal nos3 release in gastric cancer. Cancer Cell Int 24:184. doi: 10.1186/s12935-024-03359-5

30. Zhang Y, Chen L, Ye X, Wu Z, Zhang Z, Sun B, Fu H, Fu C, Liang X and Jiang H (2021) Expression and mechanism of exosome-mediated A FOXM1 related long noncoding RNA in gastric cancer. J Nanobiotechnology 19:133. doi: 10.1186/s12951-021-00873-w

31. Luo J, Jiang L, He C, Shi M, Yang ZY, Shi M, Lu S, Li C, Zhang J, Yan M, Zhu ZG and Yan C (2023) Exosomal hsa-let-7g-3p and hsa-miR-10395-3p derived from peritoneal lavage predict peritoneal metastasis and the efficacy of neoadjuvant intraperitoneal and systemic chemotherapy in patients with gastric cancer. Gastric Cancer 26:364-378. doi: 10.1007/s10120-023-01368-3

32. Wei L, Sun J, Zhang N, Zheng Y, Wang X, Lv L, Liu J, Xu Y, Shen Y and Yang M (2020) Noncoding RNAs in gastric cancer: implications for drug resistance. Mol Cancer 19:62. doi: 10.1186/s12943-020-01185-7

33. Shang Z, Luo Z, Wang Y, Liu Q, Xin Y, Zhang M, Li X, Zeng S, Yu L, Zhang X and Zhang Y (2023) CircHIPK3 contributes to cisplatin resistance in gastric cancer by blocking autophagy-dependent ferroptosis. J Cell Physiol 238:2407-2424. doi: 10.1002/jcp.31093

34. Jing X, Xie M, Ding K, Xu T, Fang Y, Ma P and Shu Y (2022) Exosome-transmitted miR-769-5p confers cisplatin resistance and progression in gastric cancer by targeting CASP9 and promoting the ubiquitination degradation of p53. Clin Transl Med 12:e780. doi: 10.1002/ctm2.780

35. Cao S, Fu B, Cai J, Zhang D, Wang C and Wu H (2023) Linc00852 from cisplatin-resistant gastric cancer cell-derived exosomes regulates COMMD7 to promote cisplatin resistance of recipient cells through microRNA-514a-5p. Cell Biol Toxicol 39:483-496. doi: 10.1007/s10565-021-09685-y

36. Mao X, Xu J, Wang W, Liang C, Hua J, Liu J, Zhang B, Meng Q, Yu X and Shi S (2021) Crosstalk between cancer-associated fibroblasts and immune cells in the tumor microenvironment: new findings and future perspectives. Mol Cancer 20:131. doi: 10.1186/s12943-021-01428-1

37. Zhang H, Deng T, Liu R, Ning T, Yang H, Liu D, Zhang Q, Lin D, Ge S, Bai M, Wang X, Zhang L, Li H, Yang Y, Ji Z, Wang H, Ying G and Ba Y (2020) CAF secreted miR-522 suppresses ferroptosis and promotes acquired chemo-resistance in gastric cancer. Mol Cancer 19:43. doi: 10.1186/s12943-020-01168-8

38. Heidrich I and Pantel K (2022) Liquid biopsy: blood-based analyses of circulating cell-free DNA in xenografts. EMBO Mol Med 14:e16326. doi: 10.15252/emmm.202216326

39. Yu D, Li Y, Wang M, Gu J, Xu W, Cai H, Fang X and Zhang X (2022) Exosomes as a new frontier of cancer liquid biopsy. Mol Cancer 21:56. doi: 10.1186/s12943-022-01509-9

40. Cai ZR, Zheng YQ, Hu Y, Ma MY, Wu YJ, Liu J, Yang LP, Zheng JB, Tian T, Hu PS, Liu ZX, Zhang L, Xu RH and Ju HQ (2025) Construction of exosome non-coding RNA feature for non-invasive, early detection of gastric cancer patients by machine learning: a multi-cohort study. Gut. doi: 10.1136/gutjnl-2024-333522

41. Guo T, Tang XH, Gao XY, Zhou Y, Jin B, Deng ZQ, Hu Y, Xing XF, Li ZY and Ji JF (2022) A liquid biopsy signature of circulating exosome-derived mRNAs, miRNAs and lncRNAs predict therapeutic efficacy to neoadjuvant chemotherapy in patients with advanced gastric cancer. Mol Cancer 21:216. doi: 10.1186/s12943-022-01684-9

42. Guo X, Gao Y, Song Q, Wei J, Wu J, Dong J, Chen L, Xu S, Wu D, Yang X, Chen L, Li X, Ji G, Lv X and Wei B (2023) Early assessment of circulating exosomal lncRNA-GC1 for monitoring neoadjuvant chemotherapy response in gastric cancer. Int J Surg 109:1094-1104. doi: 10.1097/JS9.0000000000000249

43. Shin K, Kim J, Park SJ, Lee MA, Park JM, Choi MG, Kang D, Song KY, Lee HH, Seo HS, Lee SH, Kim B, Kim O, Park J, Kang N and Kim IH (2023) Prognostic value of soluble PD-L1 and exosomal PD-L1 in advanced gastric cancer patients receiving systemic chemotherapy. Sci Rep 13:6952. doi: 10.1038/s41598-023-33128-9

44. Zhao R, Zhang Y, Zhang X, Yang Y, Zheng X, Li X, Liu Y and Zhang Y (2018) Exosomal long noncoding RNA HOTTIP as potential novel diagnostic and prognostic biomarker test for gastric cancer. Mol Cancer 17:68. doi: 10.1186/s12943-018-0817-x

45. Ruivo CF, Adem B, Silva M and Melo SA (2017) The Biology of Cancer Exosomes: Insights and New Perspectives. Cancer Res 77:6480-6488. doi: 10.1158/0008-5472.CAN-17-0994

46. Lin Y, Huang K, Cai Z, Chen Y, Feng L, Gao Y, Zheng W, Fan X, Qiu G, Zhuang J and Feng S (2022) A Novel Exosome-Relevant Molecular Classification Uncovers Distinct Immune Escape Mechanisms and Genomic Alterations in Gastric Cancer. Front Pharmacol 13:884090. doi: 10.3389/fphar.2022.884090

47. Mockler MB, Conroy MJ and Lysaght J (2014) Targeting T cell immunometabolism for cancer immunotherapy; understanding the impact of the tumor microenvironment. Front Oncol 4:107. doi: 10.3389/fonc.2014.00107

48. Shen Y, Lin J, Jiang T, Shen X, Li Y, Fu Y, Xu P, Fang L, Chen Z, Huang H, Xia Y, Xu Z and Wang L (2025) GC-derived exosomal circMAN1A2 promotes cancer progression and suppresses T-cell antitumour immunity by inhibiting FBXW11-mediated SFPQ degradation. J Exp Clin Cancer Res 44:24. doi: 10.1186/s13046-025-03288-9

49. Liu J, Wu S, Zheng X, Zheng P, Fu Y, Wu C, Lu B, Ju J and Jiang J (2020) Immune suppressed tumor microenvironment by exosomes derived from gastric cancer cells via modulating immune functions. Sci Rep 10:14749. doi: 10.1038/s41598-020-71573-y

50. Shen DD, Pang JR, Bi YP, Zhao LF, Li YR, Zhao LJ, Gao Y, Wang B, Wang N, Wei L, Guo H, Liu HM and Zheng YC (2022) LSD1 deletion decreases exosomal PD-L1 and restores T-cell response in gastric cancer. Mol Cancer 21:75. doi: 10.1186/s12943-022-01557-1

51. Liu F, Bu Z, Zhao F and Xiao D (2018) Increased T-helper 17 cell differentiation mediated by exosome-mediated microRNA-451 redistribution in gastric cancer infiltrated T cells. Cancer Sci 109:65-73. doi: 10.1111/cas.13429

52. Li J, Sun L, Chen Y, Zhu J, Shen J, Wang J, Gu Y, Zhang G, Wang M, Shi T and Chen W (2022) Gastric cancer-derived exosomal miR-135b-5p impairs the function of Vgamma9Vdelta2 T cells by targeting specificity protein 1. Cancer Immunol Immunother 71:311-325. doi: 10.1007/s00262-021-02991-8

53. Pei X, Zhang SL, Qiu BQ, Zhang PF, Liu TS and Wang Y (2024) Cancer Cell Secreted Legumain Promotes Gastric Cancer Resistance to Anti-PD-1 Immunotherapy by Enhancing Macrophage M2 Polarization. Pharmaceuticals (Basel) 17. doi: 10.3390/ph17070951

54. Ye Z, Yi J, Jiang X, Shi W, Xu H, Cao H, Qin L, Liu L, Wang T, Ma Z and Jiao Z (2025) Gastric cancer-derived exosomal let-7 g-5p mediated by SERPINE1 promotes macrophage M2 polarization and gastric cancer progression. J Exp Clin Cancer Res 44:2. doi: 10.1186/s13046-024-03269-4

55. Qiu S, Xie L, Lu C, Gu C, Xia Y, Lv J, Xuan Z, Fang L, Yang J, Zhang L, Li Z, Wang W, Xu H, Li B and Xu Z (2022) Gastric cancer-derived exosomal miR-519a-3p promotes liver metastasis by inducing intrahepatic M2-like macrophage-mediated angiogenesis. J Exp Clin Cancer Res 41:296. doi: 10.1186/s13046-022-02499-8

56. Yi J, Ye Z, Xu H, Zhang H, Cao H, Li X, Wang T, Dong C, Du Y, Dong S and Zhou W (2024) EGCG targeting STAT3 transcriptionally represses PLXNC1 to inhibit M2 polarization mediated by gastric cancer cell-derived exosomal miR-92b-5p. Phytomedicine 135:156137. doi: 10.1016/j.phymed.2024.156137

57. Zhang Y, Wang X, Liu W, Lei T, Qiao T, Feng W and Song W (2024) CircGLIS3 promotes gastric cancer progression by regulating the miR-1343-3p/PGK1 pathway and inhibiting vimentin phosphorylation. J Transl Med 22:251. doi: 10.1186/s12967-023-04625-2

58. Ma B, Wang J and Yusufu P (2023) Tumor-derived exosome ElNF1-AS1 affects the progression of gastric cancer by promoting M2 polarization of macrophages. Environ Toxicol 38:2228-2239. doi: 10.1002/tox.23862

59. Xiao H, Fu J, Liu R, Yan L, Zhou Z and Yuan J (2024) Gastric cancer cell-derived exosomal miR-541-5p induces M2 macrophage polarization through DUSP3/JAK2/STAT3 pathway. BMC Cancer 24:957. doi: 10.1186/s12885-024-12672-1

60. Liu K, Wang H, Zhou J, Zhu S, Ma M, Xiao H and Ding Y (2024) HMGB1 in exosomes derived from gastric cancer cells induces M2-like macrophage polarization by inhibiting the NF-kappaB signaling pathway. Cell Biol Int 48:334-346. doi: 10.1002/cbin.12110

61. Wang K, Cai R, Fei S, Chen X, Feng S, Zhang L, Liu H, Zhang Z, Song J and Zhou R (2023) Melatonin enhances anti-tumor immunity by targeting macrophages PD-L1 via exosomes derived from gastric cancer cells. Mol Cell Endocrinol 568-569:111917. doi: 10.1016/j.mce.2023.111917

62. Wang F, Li B, Wei Y, Zhao Y, Wang L, Zhang P, Yang J, He W, Chen H, Jiao Z and Li Y (2018) Tumor-derived exosomes induce PD1(+) macrophage population in human gastric cancer that promotes disease progression. Oncogenesis 7:41. doi: 10.1038/s41389-018-0049-3

63. Kim W, Chu TH, Nienhuser H, Jiang Z, Del Portillo A, Remotti HE, White RA, Hayakawa Y, Tomita H, Fox JG, Drake CG and Wang TC (2021) PD-1 Signaling Promotes Tumor-Infiltrating Myeloid-Derived Suppressor Cells and Gastric Tumorigenesis in Mice. Gastroenterology 160:781-796. doi: 10.1053/j.gastro.2020.10.036

64. Li H, Chen X, Zheng S, Han B, Zhang X, Zheng X, Lu Y, Sun Q, Hu X and Wu J (2024) The expansion of MDSCs induced by exosomal PD-L1 promotes the progression of gastric cancer. J Transl Med 22:821. doi: 10.1186/s12967-024-05611-y

65. Powell DR and Huttenlocher A (2016) Neutrophils in the Tumor Microenvironment. Trends Immunol 37:41-52. doi: 10.1016/j.it.2015.11.008

66. Zhang X, Shi H, Yuan X, Jiang P, Qian H and Xu W (2018) Tumor-derived exosomes induce N2 polarization of neutrophils to promote gastric cancer cell migration. Mol Cancer 17:146. doi: 10.1186/s12943-018-0898-6

67. Shi Y, Zhang J, Mao Z, Jiang H, Liu W, Shi H, Ji R, Xu W, Qian H and Zhang X (2020) Extracellular Vesicles From Gastric Cancer Cells Induce PD-L1 Expression on Neutrophils to Suppress T-Cell Immunity. Front Oncol 10:629. doi: 10.3389/fonc.2020.00629

68. Cui JX, Xu XH, He T, Liu JJ, Xie TY, Tian W and Liu JY (2023) L-kynurenine induces NK cell loss in gastric cancer microenvironment via promoting ferroptosis. J Exp Clin Cancer Res 42:52. doi: 10.1186/s13046-023-02629-w

69. Qin J, Yang J, Cui H, Feng C and Liu A (2025) Exosomal miR-552-5p Regulates the Role of NK Cells in EMT of Gastric Cancer via the PD-1/PD-L1 Axis. J Cancer 16:406-416. doi: 10.7150/jca.102360

70. Wang Y, Zhang J, Shi H, Wang M, Yu D, Fu M, Qian Y, Zhang X, Ji R, Wang S, Gu J and Zhang X (2024) M2 Tumor-Associated Macrophages-Derived Exosomal MALAT1 Promotes Glycolysis and Gastric Cancer Progression. Adv Sci (Weinh) 11:e2309298. doi: 10.1002/advs.202309298

71. Zheng P, Luo Q, Wang W, Li J, Wang T, Wang P, Chen L, Zhang P, Chen H, Liu Y, Dong P, Xie G, Ma Y, Jiang L, Yuan X and Shen L (2018) Tumor-associated macrophages-derived exosomes promote the migration of gastric cancer cells by transfer of functional Apolipoprotein E. Cell Death Dis 9:434. doi: 10.1038/s41419-018-0465-5

72. Weizman N, Krelin Y, Shabtay-Orbach A, Amit M, Binenbaum Y, Wong RJ and Gil Z (2014) Macrophages mediate gemcitabine resistance of pancreatic adenocarcinoma by upregulating cytidine deaminase. Oncogene 33:3812-9. doi: 10.1038/onc.2013.357

73. Qu B, Liu J, Peng Z, Xiao Z, Li S, Wu J, Li S and Luo J (2024) Macrophages enhance cisplatin resistance in gastric cancer through the transfer of circTEX2. J Cell Mol Med 28:e18070. doi: 10.1111/jcmm.18070

74. Yu D, Chang Z, Liu X, Chen P, Zhang H and Qin Y (2023) Macrophage-derived exosomes regulate gastric cancer cell oxaliplatin resistance by wrapping circ 0008253. Cell Cycle 22:705-717. doi: 10.1080/15384101.2022.2146839

75. Xin L, Zhou LQ, Liu C, Zeng F, Yuan YW, Zhou Q, Li SH, Wu Y, Wang JL, Wu DZ and Lu H (2021) Transfer of LncRNA CRNDE in TAM-derived exosomes is linked with cisplatin resistance in gastric cancer. EMBO Rep 22:e52124. doi: 10.15252/embr.202052124

76. Cui HY, Rong JS, Chen J, Guo J, Zhu JQ, Ruan M, Zuo RR, Zhang SS, Qi JM and Zhang BH (2021) Exosomal microRNA-588 from M2 polarized macrophages contributes to cisplatin resistance of gastric cancer cells. World J Gastroenterol 27:6079-6092. doi: 10.3748/wjg.v27.i36.6079

77. Hedrick CC and Malanchi I (2022) Neutrophils in cancer: heterogeneous and multifaceted. Nat Rev Immunol 22:173-187. doi: 10.1038/s41577-021-00571-6

78. Zhang J, Yu D, Ji C, Wang M, Fu M, Qian Y, Zhang X, Ji R, Li C, Gu J and Zhang X (2024) Exosomal miR-4745-5p/3911 from N2-polarized tumor-associated neutrophils promotes gastric cancer metastasis by regulating SLIT2. Mol Cancer 23:198. doi: 10.1186/s12943-024-02116-6

79. Mensurado S, Blanco-Dominguez R and Silva-Santos B (2023) The emerging roles of gammadelta T cells in cancer immunotherapy. Nat Rev Clin Oncol 20:178-191. doi: 10.1038/s41571-022-00722-1

80. Veerman RE, Gucluler Akpinar G, Eldh M and Gabrielsson S (2019) Immune Cell-Derived Extracellular Vesicles - Functions and Therapeutic Applications. Trends Mol Med 25:382-394. doi: 10.1016/j.molmed.2019.02.003

81. Li Z, Suo B, Long G, Gao Y, Song J, Zhang M, Feng B, Shang C and Wang D (2020) Exosomal miRNA-16-5p Derived From M1 Macrophages Enhances T Cell-Dependent Immune Response by Regulating PD-L1 in Gastric Cancer. Front Cell Dev Biol 8:572689. doi: 10.3389/fcell.2020.572689

82. Song J, Xu X, He S, Wang N, Bai Y, Li B and Zhang S (2022) Exosomal hsa_circ_0017252 attenuates the development of gastric cancer via inhibiting macrophage M2 polarization. Hum Cell 35:1499-1511. doi: 10.1007/s13577-022-00739-9

83. Dolatshahi M, Bahrami AR, Sheikh QI, Ghanbari M and Matin MM (2024) Gastric cancer and mesenchymal stem cell-derived exosomes: from pro-tumorigenic effects to anti-cancer vehicles. Arch Pharm Res 47:1-19. doi: 10.1007/s12272-023-01477-8

84. Li Y, Tian L, Zhao T and Zhang J (2023) A nanotherapeutic system for gastric cancer suppression by synergistic chemotherapy and immunotherapy based on iPSCs and DCs exosomes. Cancer Immunol Immunother 72:1673-1683. doi: 10.1007/s00262-022-03355-6

85. Zhu D, Zhang T, Li Y, Huang C, Suo M, Xia L, Xu Y, Li G and Tang BZ (2022) Tumor-derived exosomes co-delivering aggregation-induced emission luminogens and proton pump inhibitors for tumor glutamine starvation therapy and enhanced type-I photodynamic therapy. Biomaterials 283:121462. doi: 10.1016/j.biomaterials.2022.121462

86. You B, Jin C, Zhang J, Xu M, Xu W, Sun Z and Qian H (2022) MSC-Derived Extracellular Vesicle-Delivered L-PGDS Inhibit Gastric Cancer Progression by Suppressing Cancer Cell Stemness and STAT3 Phosphorylation. Stem Cells Int 2022:9668239. doi: 10.1155/2022/9668239

87. Zhang Q, Zhang H, Ning T, Liu D, Deng T, Liu R, Bai M, Zhu K, Li J, Fan Q, Ying G and Ba Y (2020) Exosome-Delivered c-Met siRNA Could Reverse Chemoresistance to Cisplatin in Gastric Cancer. Int J Nanomedicine 15:2323-2335. doi: 10.2147/IJN.S231214

88. Wang J, Li W, Lu Z, Zhang L, Hu Y, Li Q, Du W, Feng X, Jia H and Liu BF (2017) The use of RGD-engineered exosomes for enhanced targeting ability and synergistic therapy toward angiogenesis. Nanoscale 9:15598-15605. doi: 10.1039/c7nr04425a

89. Guo Z, Zhang Y, Xu W, Zhang X and Jiang J (2022) Engineered exosome-mediated delivery of circDIDO1 inhibits gastric cancer progression via regulation of MiR-1307-3p/SOCS2 Axis. J Transl Med 20:326. doi: 10.1186/s12967-022-03527-z

90. Zhang H, Wang Y, Bai M, Wang J, Zhu K, Liu R, Ge S, Li J, Ning T, Deng T, Fan Q, Li H, Sun W, Ying G and Ba Y (2018) Exosomes serve as nanoparticles to suppress tumor growth and angiogenesis in gastric cancer by delivering hepatocyte growth factor siRNA. Cancer Sci 109:629-641. doi: 10.1111/cas.13488

91. Kimura Y, Ohzawa H, Miyato H, Kaneko Y, Kuchimaru T, Takahashi R, Yamaguchi H, Kurashina K, Saito S, Hosoya Y, Lefor AK, Sata N and Kitayama J (2023) Intraperitoneal transfer of microRNA-29b-containing small extracellular vesicles can suppress peritoneal metastases of gastric cancer. Cancer Sci 114:2939-2950. doi: 10.1111/cas.15793

92. Wu P, Wang M, Jin C, Li L, Tang Y, Wang Z, Wang X, Xu W and Qian H (2024) Highly Efficient Delivery of Novel MiR-13896 by Human Umbilical Cord Mesenchymal Stem Cell-Derived Small Extracellular Vesicles Inhibits Gastric Cancer Progression by Targeting ATG2A-Mediated Autophagy. Biomater Res 28:0119. doi: 10.34133/bmr.0119

93. Wang X, Zhang H, Bai M, Ning T, Ge S, Deng T, Liu R, Zhang L, Ying G and Ba Y (2018) Exosomes Serve as Nanoparticles to Deliver Anti-miR-214 to Reverse Chemoresistance to Cisplatin in Gastric Cancer. Mol Ther 26:774-783. doi: 10.1016/j.ymthe.2018.01.001

94. Morgan E, Arnold M, Camargo MC, Gini A, Kunzmann AT, Matsuda T, Meheus F, Verhoeven RHA, Vignat J, Laversanne M, Ferlay J and Soerjomataram I (2022) The current and future incidence and mortality of gastric cancer in 185 countries, 2020-40: A population-based modelling study. EClinicalMedicine 47:101404. doi: 10.1016/j.eclinm.2022.101404

95. Alsina M, Arrazubi V, Diez M and Tabernero J (2023) Current developments in gastric cancer: from molecular profiling to treatment strategy. Nat Rev Gastroenterol Hepatol 20:155-170. doi: 10.1038/s41575-022-00703-w

96. Liu Y, Li C, Lu Y, Liu C and Yang W (2022) Tumor microenvironment-mediated immune tolerance in development and treatment of gastric cancer. Front Immunol 13:1016817. doi: 10.3389/fimmu.2022.1016817

97. Yang S, Wei S and Wei F (2024) Extracellular vesicles mediated gastric cancer immune response: tumor cell death or immune escape? Cell Death Dis 15:377. doi: 10.1038/s41419-024-06758-8

98. Zhang H, Yang M, Wu X, Li Q, Li X, Zhao Y, Du F, Chen Y, Wu Z, Xiao Z, Shen J, Wen Q, Hu W, Cho CH, Chen M, Zhou Y and Li M (2021) The distinct roles of exosomes in tumor-stroma crosstalk within gastric tumor microenvironment. Pharmacol Res 171:105785. doi: 10.1016/j.phrs.2021.105785

99. Chen DS and Mellman I (2013) Oncology meets immunology: the cancer-immunity cycle. Immunity 39:1-10. doi: 10.1016/j.immuni.2013.07.012

100. Chong X, Madeti Y, Cai J, Li W, Cong L, Lu J, Mo L, Liu H, He S, Yu C, Zhou Z, Wang B, Cao Y, Wang Z, Shen L, Wang Y and Zhang X (2024) Recent developments in immunotherapy for gastrointestinal tract cancers. J Hematol Oncol 17:65. doi: 10.1186/s13045-024-01578-x

101. Tang L, Zhang W, Qi T, Jiang Z and Tang D (2025) Exosomes play a crucial role in remodeling the tumor microenvironment and in the treatment of gastric cancer. Cell Commun Signal 23:82. doi: 10.1186/s12964-024-02009-7
