# Supplementary material for: Recent progress in the study of exosomes in the gastric cancer immune microenvironment
Source: Front Immunol. 2025 Jul 23;16:1595124. doi: 10.3389/fimmu.2025.1595124 (PMC12325315; doi:10.3389/fimmu.2025.1595124)
Supplement: Supplementary file 1 [file DataSheet1.zip › 批注文件/exosome GC 2.0 LeiZhu.docx]

**Recent progress in the study of exosomes in the gastric cancer immune microenvironment**

**Abstract**

Gastric cancer (GC) ranks among the most prevalent forms of cancer and contributes significantly to cancer-related mortality. There exists a pressing need to investigate novel approaches for GC management to improve diagnostic methods, therapeutic interventions, and patient outcomes. Exosomes are nanoscale extracellular vesicles (EVs) derived from various cell types that carry a diverse range of biomolecular cargo, including DNA, RNA, proteins, lipids, and other bioactive constituents. They play significant roles in GC pathogenesis and tumor microenvironment (TME) modulation. Exosomes derived from cancer cells can enhance tumor progression, transform the TME, and modulate immune responses. Immune cell-derived exosomes can similarly modulate immune functions and the TME. Immunotherapy represents a GC treatment breakthrough and is expected to show efficacy when combined with exosome-targeted therapy. Abundant research has demonstrated that exosomes are crucial for tumor growth, immune evasion, immune microenvironment reconfiguration, and immunotherapy efficacy in GC. This review describes the role of exosomes in the GC microenvironment, focusing on the mechanisms by which exosomes regulate immune responses to GC, and summarizes the current status of and challenges in the development of exosome-based diagnostics and immunotherapy for GC.

**Introduction**

Gastric cancer (GC) is the fourth-most common cause of cancer-related deaths and the fifth-most common cancer type globally [1]. Factors affecting the development of GC include genetic polymorphisms, environmental exposures, age, sex, and infection with *Helicobacter pylori* [2]. Due to the low proportion of early-stage diagnoses and lack of definite clinical symptoms, GC is commonly detected in the advanced metastatic phase, where the 5-year survival rate is only approximately 32% [3]. Effective treatment options for GC include surgery, radiotherapy, chemotherapy, targeted therapy, and immunotherapy, but even after surgery, approximately 60% of patients experience local recurrence or distant metastasis [4]. As a result, the exploration of new, robust biomarkers and therapeutic strategies is crucial for improving the prognosis and quality of life of GC patients.

The prognosis of GC patients and their responses to immunotherapy are impacted considerably by morphological and molecular heterogeneity in the tumor microenvironment (TME). Immunomodulatory cells in the GC TME include regulatory T cells, tumor-infiltrating myeloid-derived suppressor cells (MDSCs), tumor-associated macrophages (TAMs), and natural killer (NK) cells [5]. Immune cells can also interact with cancer cells to influence the onset and progression of cancer.

Exosomes are a type of extracellular vesicle (EV) with diameters of 30–150 nm. They are secreted by virtually all types of cells and can be stably present in a variety of biological fluids [6]. Exosomes are composed of a lipid bilayer that contains transmembrane proteins and carry cytoplasmic proteins, lipids, and nucleic acids; delivery of this cargo can mediate local and distant cell communication under physiological and pathological conditions [7]. Several studies have shown that exosome-mediated transport of bioactive signaling molecules in the TME has diagnostic and therapeutic functions [8]. Indeed, numerous lines of investigation have demonstrated that the delivery of exosome cargo is vital for GC proliferation, metastasis, drug resistance, immune response, and treatment [9]. Exosomes have also attracted attention in the field because they are more stable than circulating proteins and hormones and can serve as early biomarkers for cancer detection and disease progression [10]. In addition, improvements in exosome engineering technology have led to the development of targeted exosomes, a promising way to deliver anti-neoplastic therapies [11]. Indeed, due to their biocompatibility, low immunogenicity, and ability to transport biomolecules between cells and cross biological barriers, they are suitable for the targeted delivery of various therapeutic modalities such as small molecules, siRNA, and miRNA [12].

The tumorigenesis and progression of GC are intimately associated with immune cells and other types of mesenchymal stromal cells (MSCs), cytokines, and exosomes in the TME [13]. Exosomes derived from cancer cells or tumor-associated immune cells can carry factors that suppress immune cell activity and help tumors evade immune surveillance [14]. Therefore, it is important to investigate the mechanisms by which exosomes regulate the immune microenvironment of GC. To this end, this review aims to summarize the mechanisms of exosome-mediated GC development, focusing on recent studies of exosome-mediated immune escape. In addition, tumor immunotherapies, which consist of immune checkpoint inhibitors, cellular immunotherapy, and therapeutic cancer vaccines, have drawn substantial attention within the field. Identifying key targets and elucidating the molecular mechanisms associated with GC immunity will improve our understanding of GC pathogenesis and augment immunotherapy efficacy [15]. Thus, we have reviewed the role of exosomes in GC immune responses, as well as recent advances in exosome-targeted treatments. Together, these studies provide a basis for potential GC treatment strategies.

**1. The role of exosomes in GC**

Abundant evidence indicates that exosomes are intricately linked to GC tumorigenesis, progression, metastasis, immune escape, and drug resistance through the delivery of functional biomolecules [16]. Cancer initiation and development are generally influenced by the function of cancer cells or cells in the microenvironment. The bioactive substances carried by exosomes, including exosomal proteins, miRNAs, long non-coding RNAs (lncRNAs), and circular RNAs (circRNAs), are involved in many important processes [17] as described below. Recent studies have revealed that exosomal circ_0001789 can modulate PAK2 expression by sequestering miR-140-3p, which in turn promotes the malignant development of GC [18]. In addition, studies have shown that LINC00853 derived from GC cell exosomes promotes the epithelial-mesenchymal transition (EMT) via the MAP17/PDZK1/AKT signaling pathway, which in turn promotes GC progression [19]. Other studies have found that tumor-derived exosomal LINC01480 can up-regulate the expression of VCAM1 through competitive binding with miR-204-5p, thereby promoting the proliferation, migration, invasion, and EMT of GC cells, and suggesting that LINC01480 may be a potential therapeutic target for GC [20]. Another study showed that exosomal circSHKBP1 sequesters miR-582-3p to increase HUR expression and enhance the stability of VEGF mRNA. Furthermore, circSHKBP1 directly binds to heat shock protein 90 (HSP90), which blocks the interaction of STUB1 with HSP90, inhibits HSP90 ubiquitination, and contributes to GC development [21]. In addition, exosomal circRELL1 can inhibit the malignant transformation of GC by regulating autophagy activation in GC cells, resulting in the sequestration of miR-637 and indirectly regulating the expression of ephrin B3 (EPHB3) [22]. Other studies have shown that GC-derived exosomes containing miR-15b-3p can contribute to the development of GC via the DYNLT1/caspase-3/caspase-9 axis [23]. Moreover, exosome-derived LINC01559 activates the PI3K/AKT pathway and accelerates GC progression by up-regulating PGK1 and down-regulating PTEN [24]. Finally, it has been demonstrated that exosomal circSTAU2 may function as a tumor suppressor, curbing the advancement of GC via the miR-589/CAPZA1 pathway [25].

Exosomes also have the capacity to facilitate the migration/metastasis of GC cells to local or distant tissues and organs. At initial diagnosis, over half of GC patients have detectable lymph node metastases, which frequently results in the development of distant metastasis and a poor prognosis [26]. GC cell-derived exosomal lncAKR1C2 encodes the micro-protein pep-AKR1C2, which enhances CPT1A expression through the regulation of YAP1 phosphorylation. It also improves the tube-forming ability and migratory capacity of lymphatic endothelial cells, thereby facilitating lymphangiogenesis and promoting tumor lymphatic metastasis *in vivo* [27]. The establishment of pre-metastatic niches (PMN) in distant organs is critical for tumor metastasis [28]. Studies have shown that NOS3 originating from GC cell-derived exosomes can increase the concentration of nitric oxide within human umbilical vein endothelial cells, triggering angiogenesis, facilitating the formation of liver PMNs, and augmenting GC hepatic metastasis [29]. Furthermore, exosomes released from GC cells promote the growth and metastatic progression of GC via the delivery of FRLnc1, a viable target for GC diagnosis and treatment [30]. Other studies have shown that exosomes from GC peritoneal lavage fluid are rich in numerous miRNAs associated with peritoneal metastasis, such as let-7g-3p and miR-10395-3p, which can be used as biomarkers of peritoneal metastasis and chemotherapy efficacy [31].

Exosomes also mediate intercellular crosstalk during cancer progression and enhance therapeutic resistance. Currently, cisplatin-based chemotherapy is the primary treatment choice for advanced GC patients; however, a significant number of patients exhibit cisplatin resistance due to epigenetic alterations, signaling pathway aberrations, and disruptions in cell metabolism [32]. CircHIPK3 has been shown to promote GC cisplatin resistance by obstructing ferroptosis and, when present in serum exosomes, may be a non-invasive marker of cisplatin resistance [33]. In addition, exosomal miR-769-5p from cisplatin-resistant GC cell lines imparts cisplatin resistance to recipient GC cells. It further promotes cancer progression by targeting caspase-9 and enhancing the ubiquitination and degradation of p53 [34]. Furthermore, exosomal LINC00852 originating from cisplatin-resistant GC cells also modulates COMMD7 via miR-514a-5p to promote cisplatin resistance in recipient cells [35]. Finally, cancer-associated fibroblasts (CAFs) are the primary stromal cell type in the TME [36]. These cells secrete exosomal miR-522, which inhibits cancer cell ferroptosis by targeting ALOX15 and blocking lipid peroxidation, mediating acquired chemotherapy resistance in GC [37].

Early detection and diagnosis of GC are crucial for improving patient prognosis. Liquid biopsy is a non-invasive method for detecting circulating tumor cells, circulating tumor DNA, and EVs in serum and other bodily fluids [38]. Exosomal ncRNAs have shown great promise as molecular cancer diagnostic biomarkers [39]. Indeed, 182 candidate GC biomarkers in serum exosomes have been identified via RNA sequencing, facilitating the machine learning-assisted identification of exosomal ncRNA characteristics for non-invasive, early detection of GC [40]. In addition, the characteristics of circulating exosome-derived mRNA, miRNA, and lncRNA in liquid biopsies have the potential to predict the therapeutic outcomes of neoadjuvant chemotherapy (neoCT) in advanced GC patients [41]. Still other research has demonstrated that lncRNA-GC1 derived from circulating EVs serves as an early indicator of neoCT efficacy and can predict the survival rates of GC patients undergoing this therapy [42]. Additionally, exosomal programmed death ligand 1 (PD-L1) is linked to systemic inflammatory markers, immunomodulatory cytokines, and T cells, and exosomal PD-L1 in serum may reflect an immunosuppressive state in advanced GC patients [43]. Finally, it has been demonstrated that exosomal-derived lncRNA HOTTIP may be a novel biomarker for GC diagnosis and outcome prediction [44].

**Table 1 The roles of exosomes in GC**

| **Origin** | **Target** | **Contents** | **Mechanism** | **Effect** | **Ref.** |
| --- | --- | --- | --- | --- | --- |
| **GC cells** | **GC cells** | **Circ0001789** | **Exosomal circ0001789 sponges miR-140-3p and regulates PAK2** | **Promote malignant phenotype of GC cells** | **(18)** |
| **GC cells** | **GC cells** | **Lnc00853** | **Exosomal lnc00853 regulates the MAP17/PDZK1/AKT signaling pathway** | **Promote EMT and promote GC progression** | **(19)** |
| **GC cells** | **GC cells** | **Lnc01480** | **Exosomal lnc01480 up-regulates VCAM1 expression through competitive binding with miR-204-5p** | **Promote the proliferation, migration, invasion and EMT of GC cells** | **(20)** |
| **GC cells** | **GC cells** | **CircSHKBP1** | **Exosomal circSHKBP1 regulates the miR-582-3p/HUR/VEGF axis and inhibits HSP90 degradation** | **Promote GC progression** | **(21)** |
| **GC cells** | **GC cells** | **CircRELL1** | **Exosomal circRELL1 sponges miR-637 and indirectly dysregulates EPHB3** | **Regulate autophagy activation in GC and inhibits the malignant progression of GC** | **(22)** |
| **GC cells** | **GC cells** | **MiR-15b-3p** | **Exosomal miR-15b-3p regulates the DYNLT1/ Caspase3/Caspase9 signaling pathway** | **Promote the occurrence and development of GC** | **(23)** |
| **MSCs** | **GC cells** | **Lnc01559** | **Exosomal lnc01559 activates the PI3K/AKT pathway by up-regulating PGK1 and down-regulating PTEN** | **Promote proliferation and migration of GC cells** | **(24)** |
| **GES1 cells** | **GC cells** | **CircSTAU2** | **Exosomal circSTAU2 sponges miR-589 to reduce its inhibition of CAPZA1** | **Inhibit GC progression** | **(25)** |
| **GC cells** | **Lymphatic endothelial cells** | **LncAKR1C2** | **Exosomal lncAKR1C2 promotes CPT1A expression by regulating YAP phosphorylation** | **Enhance tube formation and migration of lymphatic endothelial cells, and promote lymphangiogenesis and lymphatic metastasis in vivo** | **(27)** |
| **GC cells** | **Human umbilical vein endothelial cells** | **NOS3** | **MiR-605-3p mediates the production of exosomal NOS3** | **Induce angiogenesis, establishe liver PMN, and promote liver metastasis** | **(29)** |
| **GC cells** | **Serum** | **CircHIPK3** | **CircHIPK3 blocks autophagy dependent ferroptosis** | **Can be used as a noninvasive index to evaluate cisplatin resistance in GC** | **(31)** |
| **Cisplatin-resistant GC cells** | **GC cells** | **MiR-769-5p** | **Exosomal miR-769-5p targets caspase-9 and promote ubiquitination degradation of p53** | **Lead to cisplatin resistance in GC and promote cancer progression** | **(32)** |
| **Cisplatin-resistant GC cells** | **GC cells** | **Lnc00852** | **Exosomal lnc00852 regulates COMMD7 through miR-514a-5p** | **Lead to cisplatin resistance in GC** | **(33)** |
| **CAFs** | **GC cells** | **MiR-522** | **Exosomal miR-522 targets ALOX15 and inhibits ferroptosis** | **Mediate acquired chemotherapy resistance in GC** | **(35)** |
| **GC cells** | **Circulating EVs** | **LncGC1** | **As an early marker of therapeutic efficacy of neoCT** | | **(40)** |
| **GC cells** | **Serum** | **PD-L1** | **Reflect the immunosuppressive state of advanced GC patients** | | **(41)** |
| **GC cells** | **Serum** | **LncHOTTIP** | **As a potential diagnostic and prognostic biomarker for gastric cancer** | | **(42)** |
| **GC cells** | **Peritoneal lavage samples** | **Let-7g-3p and miR-10395-3p** | **Can be used as a biomarker to predict the effect of peritoneal metastasis and systemic chemotherapy** | | **(43)** |

**2. Exosomes can mediate GC immune escape**

**2.1. Exosomes derived from GC cells mediate immune escape** Cancer cells can regulate the immune milieu by releasing exosomes. Many studies have revealed that exosomes make critical contributions to the reconfiguration of the TME, thereby facilitating cancer cells’ escape from the immune system [45]. Therefore, exploring the molecular characteristics of exosomes is critical for improving our understanding of immune escape mechanisms in GC [46].T cell activation is central to the anti-tumor immune response [47]. Some studies have found that exosomal circMAN1A2 can promote the development of GC and inhibit T cell anti-tumor activity. Specifically, circMAN1A2 competes with FBXW11 to bind and stabilize SFPQ expression, inhibiting T cell receptor stimulation and reducing T cell anti-tumor activity [48]. Other work has demonstrated that exosomes extracted from GC cell lines can alter the function and gene expression of CD8+ T cells, increase the frequency of effector memory CD4+ T cells and MDSCs, and reduce the frequency of CD8+ T cells and NK cells. Consistent with this observation, mice injected with GC cell-derived exosomes develop an immunosuppressive pulmonary TME. Studies have shown that exosomes originating from GC cells regulate the TME by suppressing immune function [49]. Moreover, other studies have shown that LSD1 restricts T cell responses in the GC TME by triggering the aggregation of PD-L1 in GC exosomes, providing a novel target for GC immunotherapy [50]. Furthermore, exosomes facilitate the transfer of miR-451 from GC cells to infiltrating T cells, leading to an increase in T cell Th17 polarization via reduced AMPK and enhanced mTOR activity [51]. Finally, Vγ9Vδ2 T cells can effectively internalize exosomes derived from GC cells that carry miR-135b-5p. This miR impairs the function of Vγ9Vδ2 T cells by targeting specific protein 1 (SP1), inducing apoptosis, and reducing the production of the cytotoxic cytokines IFN-γ and TNF-α. Thus, targeting the exosomal miR-135b-5p/SP1 axis may improve the efficiency of Vγ9Vδ2 T cell-based GC immunotherapy [52].

TAMs, particularly M2-polarized TAMs, can be recruited and regulated by tumor-derived inflammatory cytokines and immunosuppressive metabolites, rendering them important mediators of GC tumor progression, immune escape, and therapeutic resistance [53]. Studies have demonstrated that in GC cells, increased SERPINE1 expression leads to higher levels of let-7g-5p in exosomes. In turn, exosomal let-7g-5p is transferred to and taken up by macrophages, reducing the levels of SOCS7. By disrupting the interaction between SOCS7 and STAT3, it removes the inhibitory effect on STAT3 phosphorylation, leading to STAT3 over-activation and driving M2 polarization [54]. Liver metastasis (LM) confers a poor prognosis to individuals suffering from GC. Notably, miR-519a-3p expression in exosomes from GC patients with LM is strikingly elevated compared to exosomes from GC patients without LM. Exosomal miR-519a-3p triggers the activation of the MAPK/ERK pathway by targeting DUSP2, resulting in the M2-like polarization of macrophages, facilitating the establishment of a pre-metastatic intrahepatic niche, and promoting GC-LM progression [55]. In addition, PLXNC1 inhibits SOCS7-STAT3 interactions by transferring GC cell-derived exosomal miR-92b-5p to macrophages, activating STAT3, and promoting GC cell proliferation and M2 TAM polarization [56]. In addition, exosomal circGLIS3 promotes GC metastasis and the M2-like polarization of macrophages. Mechanistically, circGLIS3 sequesters miR-1343-3p, up-regulating PGK1 expression and modulating vimentin phosphorylation to drive GC tumorigenesis [57]. Additionally, ELNF1-AS1 is highly enriched in GC-derived exosomes and targets miR-4644 to trigger PKM expression. Exosomal ELNF1-AS1 in GC exosomes can also regulate glycolysis through PKM in a HIF-1α-dependent manner, where it contributes to M2 TAM polarization and macrophage recruitment, thereby enhancing the growth and metastatic capacity of GC cells [58]. Another study demonstrated that GC cells can also induce macrophage M2 polarization through the DUSP3/JAK2/STAT3 pathway, which is mediated by exosomal miR-541-5p [59]. Moreover, the polarization of macrophages towards the M2-like phenotype is regulated by the deactivation of the NF-κB signaling pathway. This process results from the inhibition of p50 transcriptional activity via the engagement of HMGB1, present in exosomes derived from GC cells, with the transcription factor POU2F1 [60].

Melatonin (MLT) is a hormone with potential anti-tumor properties. MLT can regulate specific miRNAs in cancer-derived exosomes that modulate the levels of PD-L1 in macrophages, increasing their secretion of anti-tumoral TNF-α and CXCL10. These findings demonstrate that MLT modulates the immune TME by regulating exosomes originating from GC cells [61]. Other studies have demonstrated that GC-derived exosomes can efficiently induce the generation of PD-1-expressing TAMs. These cells can secrete substantial amounts of IL-10, which impairs the function of CD8+ T cells, thereby establishing conditions that promote the development of GC [62].

MDSCs are the principal immunosuppressive cells in the TME, and up-regulation of PD-L1 expression in the gastric epithelium can increase the number of tumor-infiltrating MDSCs [63]. Studies have revealed that exosomal PD-L1 from GC cells may promote immunosuppression by promoting MDSC clustering and proliferation by activating the IL-6/STAT3 signaling pathway [64]. Neutrophils are also important players in cancer development and progression and can promote cancer growth, metastasis, angiogenesis, and immunosuppression [65]. Some studies have shown that neutrophils can promote tumor phenotypes through tumor polarization. Specifically, exosomes derived from GC cells induce neutrophil autophagy and promote tumor activation through HMGB1/TLR4/NF-κB signaling [66]. In addition, EVs in the GC microenvironment convey HMGB1 to trigger STAT3 activation, which up-regulates PD-L1 gene expression in neutrophils, thus inhibiting T cell-mediated immunity and highlighting the multidimensional role of EVs in regulating the immunosuppressive microenvironment [67]. Furthermore, NK cells are crucial for immune homeostasis and preventing tumorigenesis; however, reductions in their efficiency have been noted in both GC tissue and peripheral blood [68]. Indeed, studies have revealed that miR-552-5p derived from GC cell exosomes can drive the progression of GC by modulating the PD-1/PD-L1 axis, which affects NK cell function and impacts GC EMT [69].

**2.2. Exosomes from immune cells mediate GC immune escape** Considerable evidence has shown that exosomes produced by immune cells can also impact the TME and are important regulators of tumor progression. In particular, the multifunctional role of M2 TAM-derived exosomes in cancer progression has been extensively studied. Studies have shown that MALAT1 from M2 TAM-derived exosomes engages with the δ-catenin protein and impedes its ubiquitination and degradation via β-TRCP. Moreover, MALAT1 sequesters miR-217-5p to up-regulate HIF-1α expression, thereby enhancing aerobic glycolysis in GC cells. These findings imply that M2 TAM-derived exosomes facilitate GC progression through MALAT1-mediated glycolytic regulation, presenting a potential target for GC treatment [70]. Moreover, TAMs are a unique group of immune cells that express ApoE in the GC microenvironment. Indeed, M2 macrophage-derived exosomes trigger the activation of the PI3K-AKT signaling pathway in recipient GC cells via ApoE, thus enhancing the migration of GC cells [71].

TAMs are abundant in the TME and can regulate chemotherapy resistance [72]. It has been shown that M2 macrophage-derived exosomes containing circTEX2 regulate the miR-145/ABCC1 axis, thereby increasing GC cell cisplatin resistance. These data suggest that exosome transfer between macrophages and cancer cells may be a robust target for reducing cisplatin resistance in GC [73]. Furthermore, circ0008253 from M2-polarized TAM-derived exosomes can be transferred from TAMs to GC cells, ultimately enhancing GC cell resistance to oxaliplatin [74]. Furthermore, the lncRNA CRNDE is enriched in exosomes derived from M2-polarized TAMs and can be transferred to GC cells. Mechanistically, CRNDE promotes NEDD4-1-mediated PTEN ubiquitination and reduces cisplatin resistance in GC [75]. Additional work demonstrated that exosomal miR-588 secreted by M2 macrophages encourages cisplatin resistance in GC cells by partially targeting CYLD [76].

Tumor-associated neutrophils (TANs) play dual roles in tumors, where N1 TANs have anti-tumor functions and N2 TANs exhibit pro-tumor activities [77]. Neutrophil-derived exosomes regulate the initiation and progression of tumors by delivering mRNA, miRNA, and piRNA molecules. Studies have shown that exosomes from N2 TANs transfer miR-47445-5p/3911 to GC cells, down-regulating the expression of SLIT2 and promoting GC metastasis [78].

**3. The role of exosomes in GC immunotherapy**

**3.1. Exosomes can augment GC immunotherapy** Many recent studies have revealed that exosome-mediated crosstalk between cancer cells and immune cells in the TME can impact the outcome of immunotherapy [15]. For example, γδ T cells play crucial roles in innate and adaptive immune surveillance and are receiving increasing attention in the context of cancer immunotherapy. Immunotherapies based on γδ T cells have shown favorable safety profiles and clinical responses in patients with a variety of cancers [79]. Indeed, studies have demonstrated that THBS1 in exosomes derived from GC cells regulates m6A modification in Vγ9Vδ2T cells and activates the RIG-I receptor signaling pathway, leading to increased Vγ9Vδ2T cell cytotoxicity toward GC cells. Thus, targeting the exosomal THBS1/m6A/RIG-I axis could be of great significance for Vγ9Vδ2T cell-based GC immunotherapy [15].

Macrophage-derived exosomes carrying ncRNAs and immune factors can promote immune activation by regulating B cells, T cells, and NK cells [80]. Studies have demonstrated that exosomes derived from M1 macrophages, which contain miR-16-5p, can initiate T cell immune responses and impede the formation of GC tumors by decreasing the expression of PD-L1 [81]. Moreover, exosomal circ_0017252 released from GC cells can efficiently suppress M2-like polarized macrophages and inhibit the invasion and malignant progression of GC cells by sequestering miR-17-5p [82].

**3.2. Exosomes can be used as delivery vehicles for GC treatment**

Although numerous cytotoxic chemotherapeutic agents, targeted therapies, and immunomodulators have demonstrated remarkable cancer treatment efficacy, challenges such as drug resistance and side effects remain, and the development of new approaches is crucial. Recent studies indicate that the loading of therapeutic agents into nanoparticles designed specifically to target GC may improve treatment outcomes and greatly reduce adverse effects [83]. For example, some studies have investigated the effects of a tumor-targeting nanosystem, in combination with chemotherapy and immunotherapy, on GC treatment and prognosis. Specifically, a tumor-targeting system based on a fusion vector of modified iPSC and DC exosomes, DOX@aiPS-DCexo, was developed and modified with an anti-PD-1 antibody. Additionally, when the chemotherapy drug doxorubicin (DOX) was loaded into the DOX@aiPS-DCexo fusion system, it was capable of specifically targeting and eliminating tumor tissues. The system also had the ability to activate and enhance a range of local immune responses and mitigate tumor-associated immunosuppression, highlighting the efficacy of combined chemotherapy and immunotherapy treatment [84]. In addition, the efficacy of aggregation-induced emission luminogen (AIEgen)-based photodynamic therapy (PDT) is constrained by cellular glutathione (GSH), the latter of which must be reduced to effectively induce oxidation within tumor cells. Consistent with this observation, studies have leveraged tumor-derived exosomes for the co-delivery of AIEgens and proton pump inhibitors in the context of tumor combination treatment. This system can restrain cell glutamine metabolism, inhibit the generation of GSH and ATP in tumor cells, improve the effect of AIEgen type I PDT, and promote immunogenic tumor death [85]. Other studies have described the development of engineered exosomes, consisting of genetically engineered adipose-derived stem cells that express the MKN45-binding peptide DE532 on their surfaces and in which 17-(dimethylaminoethyl amino)-17-demethoxygeldanamycin (17-DMAG) has been encapsulated. These targeted, 17-DMAG-loaded DE532 exosomes effectively delivered anti-cancer agents, enhancing the therapeutic responses of GC [11]. Moreover, the lipid carrier protein prostaglandin D2 synthase (L-PGDS) has been shown to inhibit GC growth. L-PGDS-loaded EVs (EVs-L-PGDS) were produced by transducing MSCs with adenovirus encoding L-PGDS. These EVs-L-PGDS decreased the expression of stem cell markers such as Oct4, Nanog, and Sox2, and blocked STAT3 phosphorylation, suppressing GC tumor development and suggesting that MSC-derived EVs can act as efficient nano-capsules [86]. Another study isolated exosomes from HEK293T cells transfected with si-c-Met (exo-si-c-Met). The authors found that exo-si-c-Met suppressed the progression of GC cells, enhanced GC cell apoptosis, and reversed cisplatin resistance [87].

In addition, exosome-mediated delivery of RNA has demonstrated remarkable cancer treatment potential. Indeed, exosome-mediated siRNA, miRNA, and anti-miRNA oligonucleotide delivery has been extensively studied in the treatment of diverse cancers; modification of these exosomes through engineering can further improve their targeting capacity and therapeutic efficacy [88]. Studies have demonstrated that circDIDO1 suppresses GC progression via the regulation of the miR-1307-3p/SOSC2 axis and that the use of RGD-modified exosomes with circDIDO1 (RGD-Exo-circDIDO1) can reduce GC occurrence and invasion. These results suggest that engineered RGD-Exo-circDIDO1 could represent a feasible nanomedicine for GC treatment [89]. Additionally, exosomes acting as nanoparticles impede tumor advancement and angiogenesis in GC by transporting hepatocyte growth factor siRNA [90]. In another study, the quantity of miR-29b within the peritoneal exosomes of patients with pre-metastases (PMs) was markedly reduced. Transduction of human bone marrow-derived MSCs with an integrated recombinant lentiviral vector encoding miR-29b confirmed that sEVs from bone marrow MSCs were effective carriers of miR-29 that could inhibit the development of PM in GC [91]. Still another study used electroporation to insert miR-13896 into hucMSC-EVs. These engineered EVs were effectively transported to tumor sites where miR-13896 specifically targets and down-regulates the ATG2A-mediated autophagy pathway, thereby significantly suppressing the growth and metastasis of GC cells [92]. Furthermore, exosomes were utilized to deliver anti-miR-214, with the aim of reversing cisplatin-based chemotherapy resistance in GC, and successfully suppressed tumor growth [93].

**Discussion**

GC poses a global healthcare challenge. By 2040, it is projected that the incidence of GC will rise by 62%, resulting in a substantial burden on public health services, costs, and patient quality of life [94]. GC patients treated with chemotherapy and surgery have a poor prognosis, and many current clinical trials of late-stage tumors are evaluating targeted agents and immunotherapies [95]. The GC TME is a highly structured ecological system containing cancer cells, immune cells, CAFs, endothelial cells, pericytes, and various other cell types. These elements work together to sustain proliferation signals, initiate invasion and metastasis, and suppress immune reactions [96]. Exosomes are important messengers between cancer cells and TME cells. Indeed, preliminary studies have shown that exosomes generated by cancer cells control the phenotypes and functions of TME cells, driving tumor growth, metastasis, and the emergence of treatment resistance. Exosomes derived from TME cells also contain a broad spectrum of bioactive molecules and have been implicated in the regulation of tumor malignancy [78].

Exosomes play crucial roles in GC-associated immune responses. Specifically, numerous studies have shown that tumor-derived exosomes affect the differentiation, proliferation, and functional regulation of various immune cell populations in the TME, including T cells, macrophages, neutrophils, and MDSCs [97]. This review focuses on the complex mechanisms by which tumor-derived exosomes regulate GC progression, the immune microenvironment, and immune escape. In addition, exosomes derived from immune cells can also regulate immune responses to GC cells and reshape the immune microenvironment through the delivery of biological molecules such as ncRNAs. Therefore, targeting exosome-secreting immune cells may represent a promising approach to improve the efficacy of GC immunotherapy [98]. Moreover, the study of exosomes in the immune microenvironment of GC tumors can facilitate the development of more personalized targeted therapy and immunotherapy regimens for GC patients; improve our understanding of the molecular mechanisms underlying GC proliferation, progression, metastasis, and treatment resistance; and reveal new diagnostic and prognostic biomarkers and potential immunotherapeutic targets.

The success of immunotherapy, which aims to reinstate normal anti-tumor immune responses, reinitiate anti-tumor immunity, and further eliminate tumor cells, demonstrates that immune escape is crucial for tumor development and growth [99]. Immunotherapy for GC has greatly improved in recent years, but there is still a paucity of targets that reliably evoke anti-tumor immunity, and challenges persist with respect to achieving precise and personalized GC immunotherapy results [100]. Due to their innate capacity for long-distance communication, outstanding biocompatibility, and ability to traverse barriers, exosomes are ideal carriers for the delivery of various molecules, including proteins, nucleic acids, chemotherapy drugs, and gene therapy molecules [83], with great potential for GC immunotherapy applications.

In the present work, we have explored the possibility of augmenting GC immunotherapy with exosome-loaded molecules and proposed the potential of exosomes as therapeutic delivery carriers. Nevertheless, the utilization of exosomes in this context is still impeded by numerous risks and challenges, including the precision and standardization of exosome extraction procedures, the need to enhance the specificity and detection efficacy of techniques such as liquid biopsy, and the absence of clinical sample verification [101]. In addition, given their immunomodulatory effects, the use of exosomes as carriers to construct targeted chemotherapy drugs may become a new approach for personalized GC treatment. Accordingly, there is an urgent need to increase the efficiency of loading drugs or antigens into exosomes and develop more convenient methods to evaluate this process.

In summary, the molecular mechanisms underlying exosome-mediated GC occurrence, development, and immune escape or immune activation should be clarified. Moreover, targeted exosomes that amplify anti-tumor immune responses should be explored so as to overcome challenges in the standardization and clinical application of exosomes. Promoting the development of novel exosome-dependent or exosome-targeted drugs will help achieve precise delivery and synergistic therapy in the context of GC immunotherapy.

**Conclusion**

In conclusion, exosomes mediate communication between GC cells and other cell types within the TME. Exosomes regulate cancer initiation, progression, metastasis, and immune responses by delivering different biomolecules. Herein, we summarized recent studies on the molecular mechanisms underlying exosome-mediated GC development. We also described the role of exosomes as biomarkers for GC diagnosis and treatment, focusing on how exosomes derived from GC or immune cells modulate GC immune escape. Moreover, exosomes are promising vectors for targeted drug delivery and have great potential in GC immunotherapy applications. More extensive studies are needed to thoroughly understand the regulatory mechanisms by which exosomes are released by cells in the immune GC microenvironment and further explore the utility of exosomes in the augmentation of immunotherapy. Together, this work will facilitate the development of novel diagnostic, prognostic, and therapeutic strategies and targets.

**References**

1. Guan WL, He Y and Xu RH (2023) Gastric cancer treatment: recent progress and future perspectives. J Hematol Oncol 16:57. doi: 10.1186/s13045-023-01451-3

2. Ajani JA, D'Amico TA, Bentrem DJ, Chao J, Cooke D, Corvera C, Das P, Enzinger PC, Enzler T, Fanta P, Farjah F, Gerdes H, Gibson MK, Hochwald S, Hofstetter WL, Ilson DH, Keswani RN, Kim S, Kleinberg LR, Klempner SJ, Lacy J, Ly QP, Matkowskyj KA, McNamara M, Mulcahy MF, Outlaw D, Park H, Perry KA, Pimiento J, Poultsides GA, Reznik S, Roses RE, Strong VE, Su S, Wang HL, Wiesner G, Willett CG, Yakoub D, Yoon H, McMillian N and Pluchino LA (2022) Gastric Cancer, Version 2.2022, NCCN Clinical Practice Guidelines in Oncology. J Natl Compr Canc Netw 20:167-192. doi: 10.6004/jnccn.2022.0008

3. Ferlay J, Colombet M, Soerjomataram I, Mathers C, Parkin DM, Pineros M, Znaor A and Bray F (2019) Estimating the global cancer incidence and mortality in 2018: GLOBOCAN sources and methods. Int J Cancer 144:1941-1953. doi: 10.1002/ijc.31937

4. Ajani JA (2005) Evolving chemotherapy for advanced gastric cancer. Oncologist 10 Suppl 3:49-58. doi: 10.1634/theoncologist.10-90003-49

5. Zhang B, Wang CM, Wu HX, Wang F, Chai YY, Hu Y, Wang BJ, Yu Z, Xia RH, Xu RH and Cao XT (2023) MFSD2A potentiates gastric cancer response to anti-PD-1 immunotherapy by reprogramming the tumor microenvironment to activate T cell response. Cancer Commun (Lond) 43:1097-1116. doi: 10.1002/cac2.12476

6. Kalra H, Drummen GP and Mathivanan S (2016) Focus on Extracellular Vesicles: Introducing the Next Small Big Thing. Int J Mol Sci 17:170. doi: 10.3390/ijms17020170

7. Tkach M and Thery C (2016) Communication by Extracellular Vesicles: Where We Are and Where We Need to Go. Cell 164:1226-1232. doi: 10.1016/j.cell.2016.01.043

8. Miron RJ, Estrin NE, Sculean A and Zhang Y (2024) Understanding exosomes: Part 2-Emerging leaders in regenerative medicine. Periodontol 2000 94:257-414. doi: 10.1111/prd.12561

9. Wang J, Zhang H, Li J, Ni X, Yan W, Chen Y and Shi T (2024) Exosome-derived proteins in gastric cancer progression, drug resistance, and immune response. Cell Mol Biol Lett 29:157. doi: 10.1186/s11658-024-00676-5

10. Jalalian SH, Ramezani M, Jalalian SA, Abnous K and Taghdisi SM (2019) Exosomes, new biomarkers in early cancer detection. Anal Biochem 571:1-13. doi: 10.1016/j.ab.2019.02.013

11. Park JH, Kim SJ, Kim OH and Kim DJ (2024) Enhanced Efficacy of Gastric Cancer Treatment through Targeted Exosome Delivery of 17-DMAG Anticancer Agent. Int J Mol Sci 25. doi: 10.3390/ijms25168762

12. Zhang Y, Li J, Gao W and Xie N (2022) Exosomes as Anticancer Drug Delivery Vehicles: Prospects and Challenges. Front Biosci (Landmark Ed) 27:293. doi: 10.31083/j.fbl2710293

13. Yu X, Zhang Y, Luo F, Zhou Q and Zhu L (2024) The role of microRNAs in the gastric cancer tumor microenvironment. Mol Cancer 23:170. doi: 10.1186/s12943-024-02084-x

14. Kalluri R (2024) The biology and function of extracellular vesicles in immune response and immunity. Immunity 57:1752-1768. doi: 10.1016/j.immuni.2024.07.009

15. Li J, Feng H, Zhu J, Yang K, Zhang G, Gu Y, Shi T and Chen W (2023) Gastric cancer derived exosomal THBS1 enhanced Vgamma9Vdelta2 T-cell function through activating RIG-I-like receptor signaling pathway in a N6-methyladenosine methylation dependent manner. Cancer Lett 576:216410. doi: 10.1016/j.canlet.2023.216410

16. Fu M, Gu J, Jiang P, Qian H, Xu W and Zhang X (2019) Exosomes in gastric cancer: roles, mechanisms, and applications. Mol Cancer 18:41. doi: 10.1186/s12943-019-1001-7

17. Wu H, Fu M, Liu J, Chong W, Fang Z, Du F, Liu Y, Shang L and Li L (2021) The role and application of small extracellular vesicles in gastric cancer. Mol Cancer 20:71. doi: 10.1186/s12943-021-01365-z

18. You J, Chen Y, Chen D, Li Y, Wang T, Zhu J, Hong Q and Li Q (2023) Circular RNA 0001789 sponges miR-140-3p and regulates PAK2 to promote the progression of gastric cancer. J Transl Med 21:83. doi: 10.1186/s12967-022-03853-2

19. Yoon JH, Byun HJ, Kim SY, Jung DH and Lee SK (2024) Exosomal LINC00853 promotes progression of gastric cancer via the MAP17/PDZK1/AKT signaling pathway. Noncoding RNA Res 9:876-886. doi: 10.1016/j.ncrna.2024.03.011

20. Zhang Y, Guo S, Mao T, Guo J, Zhang Q, Tian Z and Li X (2024) Tumor-Derived Exosomal LINC01480 Upregulates VCAM1 Expression by Acting as a Competitive Endogenous RNA of miR-204-5p to Promote Gastric Cancer Progression. ACS Biomater Sci Eng 10:550-562. doi: 10.1021/acsbiomaterials.3c00394

21. Xie M, Yu T, Jing X, Ma L, Fan Y, Yang F, Ma P, Jiang H, Wu X, Shu Y and Xu T (2020) Exosomal circSHKBP1 promotes gastric cancer progression via regulating the miR-582-3p/HUR/VEGF axis and suppressing HSP90 degradation. Mol Cancer 19:112. doi: 10.1186/s12943-020-01208-3

22. Sang H, Zhang W, Peng L, Wei S, Zhu X, Huang K, Yang J, Chen M, Dang Y and Zhang G (2022) Exosomal circRELL1 serves as a miR-637 sponge to modulate gastric cancer progression via regulating autophagy activation. Cell Death Dis 13:56. doi: 10.1038/s41419-021-04364-6

23. Wei S, Peng L, Yang J, Sang H, Jin D, Li X, Chen M, Zhang W, Dang Y and Zhang G (2020) Exosomal transfer of miR-15b-3p enhances tumorigenesis and malignant transformation through the DYNLT1/Caspase-3/Caspase-9 signaling pathway in gastric cancer. J Exp Clin Cancer Res 39:32. doi: 10.1186/s13046-019-1511-6

24. Wang L, Bo X, Yi X, Xiao X, Zheng Q, Ma L and Li B (2020) Exosome-transferred LINC01559 promotes the progression of gastric cancer via PI3K/AKT signaling pathway. Cell Death Dis 11:723. doi: 10.1038/s41419-020-02810-5

25. Zhang C, Wei G, Zhu X, Chen X, Ma X, Hu P, Liu W, Yang W, Ruan T, Zhang W, Wu C and Tao K (2023) Exosome-Delivered circSTAU2 Inhibits the Progression of Gastric Cancer by Targeting the miR-589/CAPZA1 Axis. Int J Nanomedicine 18:127-142. doi: 10.2147/IJN.S391872

26. Kim DH, Choi MG, Noh JH, Sohn TS, Bae JM and Kim S (2015) Clinical significance of skip lymph node metastasis in gastric cancer patients. Eur J Surg Oncol 41:339-45. doi: 10.1016/j.ejso.2014.09.009

27. Zhu KG, Yang J, Zhu Y, Zhu Q, Pan W, Deng S, He Y, Zuo D, Wang P, Han Y and Zhang HY (2023) The microprotein encoded by exosomal lncAKR1C2 promotes gastric cancer lymph node metastasis by regulating fatty acid metabolism. Cell Death Dis 14:708. doi: 10.1038/s41419-023-06220-1

28. Fabris L, Sato K, Alpini G and Strazzabosco M (2021) The Tumor Microenvironment in Cholangiocarcinoma Progression. Hepatology 73 Suppl 1:75-85. doi: 10.1002/hep.31410

29. Hu Y, Zang W, Feng Y, Mao Q, Chen J, Zhu Y and Xue W (2024) mir-605-3p prevents liver premetastatic niche formation by inhibiting angiogenesis via decreasing exosomal nos3 release in gastric cancer. Cancer Cell Int 24:184. doi: 10.1186/s12935-024-03359-5

30. Zhang Y, Chen L, Ye X, Wu Z, Zhang Z, Sun B, Fu H, Fu C, Liang X and Jiang H (2021) Expression and mechanism of exosome-mediated A FOXM1 related long noncoding RNA in gastric cancer. J Nanobiotechnology 19:133. doi: 10.1186/s12951-021-00873-w

31. Luo J, Jiang L, He C, Shi M, Yang ZY, Shi M, Lu S, Li C, Zhang J, Yan M, Zhu ZG and Yan C (2023) Exosomal hsa-let-7g-3p and hsa-miR-10395-3p derived from peritoneal lavage predict peritoneal metastasis and the efficacy of neoadjuvant intraperitoneal and systemic chemotherapy in patients with gastric cancer. Gastric Cancer 26:364-378. doi: 10.1007/s10120-023-01368-3

32. Wei L, Sun J, Zhang N, Zheng Y, Wang X, Lv L, Liu J, Xu Y, Shen Y and Yang M (2020) Noncoding RNAs in gastric cancer: implications for drug resistance. Mol Cancer 19:62. doi: 10.1186/s12943-020-01185-7

33. Shang Z, Luo Z, Wang Y, Liu Q, Xin Y, Zhang M, Li X, Zeng S, Yu L, Zhang X and Zhang Y (2023) CircHIPK3 contributes to cisplatin resistance in gastric cancer by blocking autophagy-dependent ferroptosis. J Cell Physiol 238:2407-2424. doi: 10.1002/jcp.31093

34. Jing X, Xie M, Ding K, Xu T, Fang Y, Ma P and Shu Y (2022) Exosome-transmitted miR-769-5p confers cisplatin resistance and progression in gastric cancer by targeting CASP9 and promoting the ubiquitination degradation of p53. Clin Transl Med 12:e780. doi: 10.1002/ctm2.780

35. Cao S, Fu B, Cai J, Zhang D, Wang C and Wu H (2023) Linc00852 from cisplatin-resistant gastric cancer cell-derived exosomes regulates COMMD7 to promote cisplatin resistance of recipient cells through microRNA-514a-5p. Cell Biol Toxicol 39:483-496. doi: 10.1007/s10565-021-09685-y

36. Mao X, Xu J, Wang W, Liang C, Hua J, Liu J, Zhang B, Meng Q, Yu X and Shi S (2021) Crosstalk between cancer-associated fibroblasts and immune cells in the tumor microenvironment: new findings and future perspectives. Mol Cancer 20:131. doi: 10.1186/s12943-021-01428-1

37. Zhang H, Deng T, Liu R, Ning T, Yang H, Liu D, Zhang Q, Lin D, Ge S, Bai M, Wang X, Zhang L, Li H, Yang Y, Ji Z, Wang H, Ying G and Ba Y (2020) CAF secreted miR-522 suppresses ferroptosis and promotes acquired chemo-resistance in gastric cancer. Mol Cancer 19:43. doi: 10.1186/s12943-020-01168-8

38. Heidrich I and Pantel K (2022) Liquid biopsy: blood-based analyses of circulating cell-free DNA in xenografts. EMBO Mol Med 14:e16326. doi: 10.15252/emmm.202216326

39. Yu D, Li Y, Wang M, Gu J, Xu W, Cai H, Fang X and Zhang X (2022) Exosomes as a new frontier of cancer liquid biopsy. Mol Cancer 21:56. doi: 10.1186/s12943-022-01509-9

40. Cai ZR, Zheng YQ, Hu Y, Ma MY, Wu YJ, Liu J, Yang LP, Zheng JB, Tian T, Hu PS, Liu ZX, Zhang L, Xu RH and Ju HQ (2025) Construction of exosome non-coding RNA feature for non-invasive, early detection of gastric cancer patients by machine learning: a multi-cohort study. Gut. doi: 10.1136/gutjnl-2024-333522

41. Guo T, Tang XH, Gao XY, Zhou Y, Jin B, Deng ZQ, Hu Y, Xing XF, Li ZY and Ji JF (2022) A liquid biopsy signature of circulating exosome-derived mRNAs, miRNAs and lncRNAs predict therapeutic efficacy to neoadjuvant chemotherapy in patients with advanced gastric cancer. Mol Cancer 21:216. doi: 10.1186/s12943-022-01684-9

42. Guo X, Gao Y, Song Q, Wei J, Wu J, Dong J, Chen L, Xu S, Wu D, Yang X, Chen L, Li X, Ji G, Lv X and Wei B (2023) Early assessment of circulating exosomal lncRNA-GC1 for monitoring neoadjuvant chemotherapy response in gastric cancer. Int J Surg 109:1094-1104. doi: 10.1097/JS9.0000000000000249

43. Shin K, Kim J, Park SJ, Lee MA, Park JM, Choi MG, Kang D, Song KY, Lee HH, Seo HS, Lee SH, Kim B, Kim O, Park J, Kang N and Kim IH (2023) Prognostic value of soluble PD-L1 and exosomal PD-L1 in advanced gastric cancer patients receiving systemic chemotherapy. Sci Rep 13:6952. doi: 10.1038/s41598-023-33128-9

44. Zhao R, Zhang Y, Zhang X, Yang Y, Zheng X, Li X, Liu Y and Zhang Y (2018) Exosomal long noncoding RNA HOTTIP as potential novel diagnostic and prognostic biomarker test for gastric cancer. Mol Cancer 17:68. doi: 10.1186/s12943-018-0817-x

45. Ruivo CF, Adem B, Silva M and Melo SA (2017) The Biology of Cancer Exosomes: Insights and New Perspectives. Cancer Res 77:6480-6488. doi: 10.1158/0008-5472.CAN-17-0994

46. Lin Y, Huang K, Cai Z, Chen Y, Feng L, Gao Y, Zheng W, Fan X, Qiu G, Zhuang J and Feng S (2022) A Novel Exosome-Relevant Molecular Classification Uncovers Distinct Immune Escape Mechanisms and Genomic Alterations in Gastric Cancer. Front Pharmacol 13:884090. doi: 10.3389/fphar.2022.884090

47. Mockler MB, Conroy MJ and Lysaght J (2014) Targeting T cell immunometabolism for cancer immunotherapy; understanding the impact of the tumor microenvironment. Front Oncol 4:107. doi: 10.3389/fonc.2014.00107

48. Shen Y, Lin J, Jiang T, Shen X, Li Y, Fu Y, Xu P, Fang L, Chen Z, Huang H, Xia Y, Xu Z and Wang L (2025) GC-derived exosomal circMAN1A2 promotes cancer progression and suppresses T-cell antitumour immunity by inhibiting FBXW11-mediated SFPQ degradation. J Exp Clin Cancer Res 44:24. doi: 10.1186/s13046-025-03288-9

49. Liu J, Wu S, Zheng X, Zheng P, Fu Y, Wu C, Lu B, Ju J and Jiang J (2020) Immune suppressed tumor microenvironment by exosomes derived from gastric cancer cells via modulating immune functions. Sci Rep 10:14749. doi: 10.1038/s41598-020-71573-y

50. Shen DD, Pang JR, Bi YP, Zhao LF, Li YR, Zhao LJ, Gao Y, Wang B, Wang N, Wei L, Guo H, Liu HM and Zheng YC (2022) LSD1 deletion decreases exosomal PD-L1 and restores T-cell response in gastric cancer. Mol Cancer 21:75. doi: 10.1186/s12943-022-01557-1

51. Liu F, Bu Z, Zhao F and Xiao D (2018) Increased T-helper 17 cell differentiation mediated by exosome-mediated microRNA-451 redistribution in gastric cancer infiltrated T cells. Cancer Sci 109:65-73. doi: 10.1111/cas.13429

52. Li J, Sun L, Chen Y, Zhu J, Shen J, Wang J, Gu Y, Zhang G, Wang M, Shi T and Chen W (2022) Gastric cancer-derived exosomal miR-135b-5p impairs the function of Vgamma9Vdelta2 T cells by targeting specificity protein 1. Cancer Immunol Immunother 71:311-325. doi: 10.1007/s00262-021-02991-8

53. Pei X, Zhang SL, Qiu BQ, Zhang PF, Liu TS and Wang Y (2024) Cancer Cell Secreted Legumain Promotes Gastric Cancer Resistance to Anti-PD-1 Immunotherapy by Enhancing Macrophage M2 Polarization. Pharmaceuticals (Basel) 17. doi: 10.3390/ph17070951

54. Ye Z, Yi J, Jiang X, Shi W, Xu H, Cao H, Qin L, Liu L, Wang T, Ma Z and Jiao Z (2025) Gastric cancer-derived exosomal let-7 g-5p mediated by SERPINE1 promotes macrophage M2 polarization and gastric cancer progression. J Exp Clin Cancer Res 44:2. doi: 10.1186/s13046-024-03269-4

55. Qiu S, Xie L, Lu C, Gu C, Xia Y, Lv J, Xuan Z, Fang L, Yang J, Zhang L, Li Z, Wang W, Xu H, Li B and Xu Z (2022) Gastric cancer-derived exosomal miR-519a-3p promotes liver metastasis by inducing intrahepatic M2-like macrophage-mediated angiogenesis. J Exp Clin Cancer Res 41:296. doi: 10.1186/s13046-022-02499-8

56. Yi J, Ye Z, Xu H, Zhang H, Cao H, Li X, Wang T, Dong C, Du Y, Dong S and Zhou W (2024) EGCG targeting STAT3 transcriptionally represses PLXNC1 to inhibit M2 polarization mediated by gastric cancer cell-derived exosomal miR-92b-5p. Phytomedicine 135:156137. doi: 10.1016/j.phymed.2024.156137

57. Zhang Y, Wang X, Liu W, Lei T, Qiao T, Feng W and Song W (2024) CircGLIS3 promotes gastric cancer progression by regulating the miR-1343-3p/PGK1 pathway and inhibiting vimentin phosphorylation. J Transl Med 22:251. doi: 10.1186/s12967-023-04625-2

58. Ma B, Wang J and Yusufu P (2023) Tumor-derived exosome ElNF1-AS1 affects the progression of gastric cancer by promoting M2 polarization of macrophages. Environ Toxicol 38:2228-2239. doi: 10.1002/tox.23862

59. Xiao H, Fu J, Liu R, Yan L, Zhou Z and Yuan J (2024) Gastric cancer cell-derived exosomal miR-541-5p induces M2 macrophage polarization through DUSP3/JAK2/STAT3 pathway. BMC Cancer 24:957. doi: 10.1186/s12885-024-12672-1

60. Liu K, Wang H, Zhou J, Zhu S, Ma M, Xiao H and Ding Y (2024) HMGB1 in exosomes derived from gastric cancer cells induces M2-like macrophage polarization by inhibiting the NF-kappaB signaling pathway. Cell Biol Int 48:334-346. doi: 10.1002/cbin.12110

61. Wang K, Cai R, Fei S, Chen X, Feng S, Zhang L, Liu H, Zhang Z, Song J and Zhou R (2023) Melatonin enhances anti-tumor immunity by targeting macrophages PD-L1 via exosomes derived from gastric cancer cells. Mol Cell Endocrinol 568-569:111917. doi: 10.1016/j.mce.2023.111917

62. Wang F, Li B, Wei Y, Zhao Y, Wang L, Zhang P, Yang J, He W, Chen H, Jiao Z and Li Y (2018) Tumor-derived exosomes induce PD1(+) macrophage population in human gastric cancer that promotes disease progression. Oncogenesis 7:41. doi: 10.1038/s41389-018-0049-3

63. Kim W, Chu TH, Nienhuser H, Jiang Z, Del Portillo A, Remotti HE, White RA, Hayakawa Y, Tomita H, Fox JG, Drake CG and Wang TC (2021) PD-1 Signaling Promotes Tumor-Infiltrating Myeloid-Derived Suppressor Cells and Gastric Tumorigenesis in Mice. Gastroenterology 160:781-796. doi: 10.1053/j.gastro.2020.10.036

64. Li H, Chen X, Zheng S, Han B, Zhang X, Zheng X, Lu Y, Sun Q, Hu X and Wu J (2024) The expansion of MDSCs induced by exosomal PD-L1 promotes the progression of gastric cancer. J Transl Med 22:821. doi: 10.1186/s12967-024-05611-y

65. Powell DR and Huttenlocher A (2016) Neutrophils in the Tumor Microenvironment. Trends Immunol 37:41-52. doi: 10.1016/j.it.2015.11.008

66. Zhang X, Shi H, Yuan X, Jiang P, Qian H and Xu W (2018) Tumor-derived exosomes induce N2 polarization of neutrophils to promote gastric cancer cell migration. Mol Cancer 17:146. doi: 10.1186/s12943-018-0898-6

67. Shi Y, Zhang J, Mao Z, Jiang H, Liu W, Shi H, Ji R, Xu W, Qian H and Zhang X (2020) Extracellular Vesicles From Gastric Cancer Cells Induce PD-L1 Expression on Neutrophils to Suppress T-Cell Immunity. Front Oncol 10:629. doi: 10.3389/fonc.2020.00629

68. Cui JX, Xu XH, He T, Liu JJ, Xie TY, Tian W and Liu JY (2023) L-kynurenine induces NK cell loss in gastric cancer microenvironment via promoting ferroptosis. J Exp Clin Cancer Res 42:52. doi: 10.1186/s13046-023-02629-w

69. Qin J, Yang J, Cui H, Feng C and Liu A (2025) Exosomal miR-552-5p Regulates the Role of NK Cells in EMT of Gastric Cancer via the PD-1/PD-L1 Axis. J Cancer 16:406-416. doi: 10.7150/jca.102360

70. Wang Y, Zhang J, Shi H, Wang M, Yu D, Fu M, Qian Y, Zhang X, Ji R, Wang S, Gu J and Zhang X (2024) M2 Tumor-Associated Macrophages-Derived Exosomal MALAT1 Promotes Glycolysis and Gastric Cancer Progression. Adv Sci (Weinh) 11:e2309298. doi: 10.1002/advs.202309298

71. Zheng P, Luo Q, Wang W, Li J, Wang T, Wang P, Chen L, Zhang P, Chen H, Liu Y, Dong P, Xie G, Ma Y, Jiang L, Yuan X and Shen L (2018) Tumor-associated macrophages-derived exosomes promote the migration of gastric cancer cells by transfer of functional Apolipoprotein E. Cell Death Dis 9:434. doi: 10.1038/s41419-018-0465-5

72. Weizman N, Krelin Y, Shabtay-Orbach A, Amit M, Binenbaum Y, Wong RJ and Gil Z (2014) Macrophages mediate gemcitabine resistance of pancreatic adenocarcinoma by upregulating cytidine deaminase. Oncogene 33:3812-9. doi: 10.1038/onc.2013.357

73. Qu B, Liu J, Peng Z, Xiao Z, Li S, Wu J, Li S and Luo J (2024) Macrophages enhance cisplatin resistance in gastric cancer through the transfer of circTEX2. J Cell Mol Med 28:e18070. doi: 10.1111/jcmm.18070

74. Yu D, Chang Z, Liu X, Chen P, Zhang H and Qin Y (2023) Macrophage-derived exosomes regulate gastric cancer cell oxaliplatin resistance by wrapping circ 0008253. Cell Cycle 22:705-717. doi: 10.1080/15384101.2022.2146839

75. Xin L, Zhou LQ, Liu C, Zeng F, Yuan YW, Zhou Q, Li SH, Wu Y, Wang JL, Wu DZ and Lu H (2021) Transfer of LncRNA CRNDE in TAM-derived exosomes is linked with cisplatin resistance in gastric cancer. EMBO Rep 22:e52124. doi: 10.15252/embr.202052124

76. Cui HY, Rong JS, Chen J, Guo J, Zhu JQ, Ruan M, Zuo RR, Zhang SS, Qi JM and Zhang BH (2021) Exosomal microRNA-588 from M2 polarized macrophages contributes to cisplatin resistance of gastric cancer cells. World J Gastroenterol 27:6079-6092. doi: 10.3748/wjg.v27.i36.6079

77. Hedrick CC and Malanchi I (2022) Neutrophils in cancer: heterogeneous and multifaceted. Nat Rev Immunol 22:173-187. doi: 10.1038/s41577-021-00571-6

78. Zhang J, Yu D, Ji C, Wang M, Fu M, Qian Y, Zhang X, Ji R, Li C, Gu J and Zhang X (2024) Exosomal miR-4745-5p/3911 from N2-polarized tumor-associated neutrophils promotes gastric cancer metastasis by regulating SLIT2. Mol Cancer 23:198. doi: 10.1186/s12943-024-02116-6

79. Mensurado S, Blanco-Dominguez R and Silva-Santos B (2023) The emerging roles of gammadelta T cells in cancer immunotherapy. Nat Rev Clin Oncol 20:178-191. doi: 10.1038/s41571-022-00722-1

80. Veerman RE, Gucluler Akpinar G, Eldh M and Gabrielsson S (2019) Immune Cell-Derived Extracellular Vesicles - Functions and Therapeutic Applications. Trends Mol Med 25:382-394. doi: 10.1016/j.molmed.2019.02.003

81. Li Z, Suo B, Long G, Gao Y, Song J, Zhang M, Feng B, Shang C and Wang D (2020) Exosomal miRNA-16-5p Derived From M1 Macrophages Enhances T Cell-Dependent Immune Response by Regulating PD-L1 in Gastric Cancer. Front Cell Dev Biol 8:572689. doi: 10.3389/fcell.2020.572689

82. Song J, Xu X, He S, Wang N, Bai Y, Li B and Zhang S (2022) Exosomal hsa_circ_0017252 attenuates the development of gastric cancer via inhibiting macrophage M2 polarization. Hum Cell 35:1499-1511. doi: 10.1007/s13577-022-00739-9

83. Dolatshahi M, Bahrami AR, Sheikh QI, Ghanbari M and Matin MM (2024) Gastric cancer and mesenchymal stem cell-derived exosomes: from pro-tumorigenic effects to anti-cancer vehicles. Arch Pharm Res 47:1-19. doi: 10.1007/s12272-023-01477-8

84. Li Y, Tian L, Zhao T and Zhang J (2023) A nanotherapeutic system for gastric cancer suppression by synergistic chemotherapy and immunotherapy based on iPSCs and DCs exosomes. Cancer Immunol Immunother 72:1673-1683. doi: 10.1007/s00262-022-03355-6

85. Zhu D, Zhang T, Li Y, Huang C, Suo M, Xia L, Xu Y, Li G and Tang BZ (2022) Tumor-derived exosomes co-delivering aggregation-induced emission luminogens and proton pump inhibitors for tumor glutamine starvation therapy and enhanced type-I photodynamic therapy. Biomaterials 283:121462. doi: 10.1016/j.biomaterials.2022.121462

86. You B, Jin C, Zhang J, Xu M, Xu W, Sun Z and Qian H (2022) MSC-Derived Extracellular Vesicle-Delivered L-PGDS Inhibit Gastric Cancer Progression by Suppressing Cancer Cell Stemness and STAT3 Phosphorylation. Stem Cells Int 2022:9668239. doi: 10.1155/2022/9668239

87. Zhang Q, Zhang H, Ning T, Liu D, Deng T, Liu R, Bai M, Zhu K, Li J, Fan Q, Ying G and Ba Y (2020) Exosome-Delivered c-Met siRNA Could Reverse Chemoresistance to Cisplatin in Gastric Cancer. Int J Nanomedicine 15:2323-2335. doi: 10.2147/IJN.S231214

88. Wang J, Li W, Lu Z, Zhang L, Hu Y, Li Q, Du W, Feng X, Jia H and Liu BF (2017) The use of RGD-engineered exosomes for enhanced targeting ability and synergistic therapy toward angiogenesis. Nanoscale 9:15598-15605. doi: 10.1039/c7nr04425a

89. Guo Z, Zhang Y, Xu W, Zhang X and Jiang J (2022) Engineered exosome-mediated delivery of circDIDO1 inhibits gastric cancer progression via regulation of MiR-1307-3p/SOCS2 Axis. J Transl Med 20:326. doi: 10.1186/s12967-022-03527-z

90. Zhang H, Wang Y, Bai M, Wang J, Zhu K, Liu R, Ge S, Li J, Ning T, Deng T, Fan Q, Li H, Sun W, Ying G and Ba Y (2018) Exosomes serve as nanoparticles to suppress tumor growth and angiogenesis in gastric cancer by delivering hepatocyte growth factor siRNA. Cancer Sci 109:629-641. doi: 10.1111/cas.13488

91. Kimura Y, Ohzawa H, Miyato H, Kaneko Y, Kuchimaru T, Takahashi R, Yamaguchi H, Kurashina K, Saito S, Hosoya Y, Lefor AK, Sata N and Kitayama J (2023) Intraperitoneal transfer of microRNA-29b-containing small extracellular vesicles can suppress peritoneal metastases of gastric cancer. Cancer Sci 114:2939-2950. doi: 10.1111/cas.15793

92. Wu P, Wang M, Jin C, Li L, Tang Y, Wang Z, Wang X, Xu W and Qian H (2024) Highly Efficient Delivery of Novel MiR-13896 by Human Umbilical Cord Mesenchymal Stem Cell-Derived Small Extracellular Vesicles Inhibits Gastric Cancer Progression by Targeting ATG2A-Mediated Autophagy. Biomater Res 28:0119. doi: 10.34133/bmr.0119

93. Wang X, Zhang H, Bai M, Ning T, Ge S, Deng T, Liu R, Zhang L, Ying G and Ba Y (2018) Exosomes Serve as Nanoparticles to Deliver Anti-miR-214 to Reverse Chemoresistance to Cisplatin in Gastric Cancer. Mol Ther 26:774-783. doi: 10.1016/j.ymthe.2018.01.001

94. Morgan E, Arnold M, Camargo MC, Gini A, Kunzmann AT, Matsuda T, Meheus F, Verhoeven RHA, Vignat J, Laversanne M, Ferlay J and Soerjomataram I (2022) The current and future incidence and mortality of gastric cancer in 185 countries, 2020-40: A population-based modelling study. EClinicalMedicine 47:101404. doi: 10.1016/j.eclinm.2022.101404

95. Alsina M, Arrazubi V, Diez M and Tabernero J (2023) Current developments in gastric cancer: from molecular profiling to treatment strategy. Nat Rev Gastroenterol Hepatol 20:155-170. doi: 10.1038/s41575-022-00703-w

96. Liu Y, Li C, Lu Y, Liu C and Yang W (2022) Tumor microenvironment-mediated immune tolerance in development and treatment of gastric cancer. Front Immunol 13:1016817. doi: 10.3389/fimmu.2022.1016817

97. Yang S, Wei S and Wei F (2024) Extracellular vesicles mediated gastric cancer immune response: tumor cell death or immune escape? Cell Death Dis 15:377. doi: 10.1038/s41419-024-06758-8

98. Zhang H, Yang M, Wu X, Li Q, Li X, Zhao Y, Du F, Chen Y, Wu Z, Xiao Z, Shen J, Wen Q, Hu W, Cho CH, Chen M, Zhou Y and Li M (2021) The distinct roles of exosomes in tumor-stroma crosstalk within gastric tumor microenvironment. Pharmacol Res 171:105785. doi: 10.1016/j.phrs.2021.105785

99. Chen DS and Mellman I (2013) Oncology meets immunology: the cancer-immunity cycle. Immunity 39:1-10. doi: 10.1016/j.immuni.2013.07.012

100. Chong X, Madeti Y, Cai J, Li W, Cong L, Lu J, Mo L, Liu H, He S, Yu C, Zhou Z, Wang B, Cao Y, Wang Z, Shen L, Wang Y and Zhang X (2024) Recent developments in immunotherapy for gastrointestinal tract cancers. J Hematol Oncol 17:65. doi: 10.1186/s13045-024-01578-x

101. Tang L, Zhang W, Qi T, Jiang Z and Tang D (2025) Exosomes play a crucial role in remodeling the tumor microenvironment and in the treatment of gastric cancer. Cell Commun Signal 23:82. doi: 10.1186/s12964-024-02009-7
