# Supplementary material for: Recent progress in the study of exosomes in the gastric cancer immune microenvironment
Source: Front Immunol. 2025 Jul 23;16:1595124. doi: 10.3389/fimmu.2025.1595124 (PMC12325315; doi:10.3389/fimmu.2025.1595124)
Supplement: Supplementary file 2 [file DataSheet2.docx]

Supplementary Material

**1 Supplementary Figure**

**Supplementary Figure 1. Mechanisms by which exosomes from immune cells in the TME inhibit GC immune response and promote cancer progression, metastasis and drug resistance.** **Created in https://BioRender.com.**

**
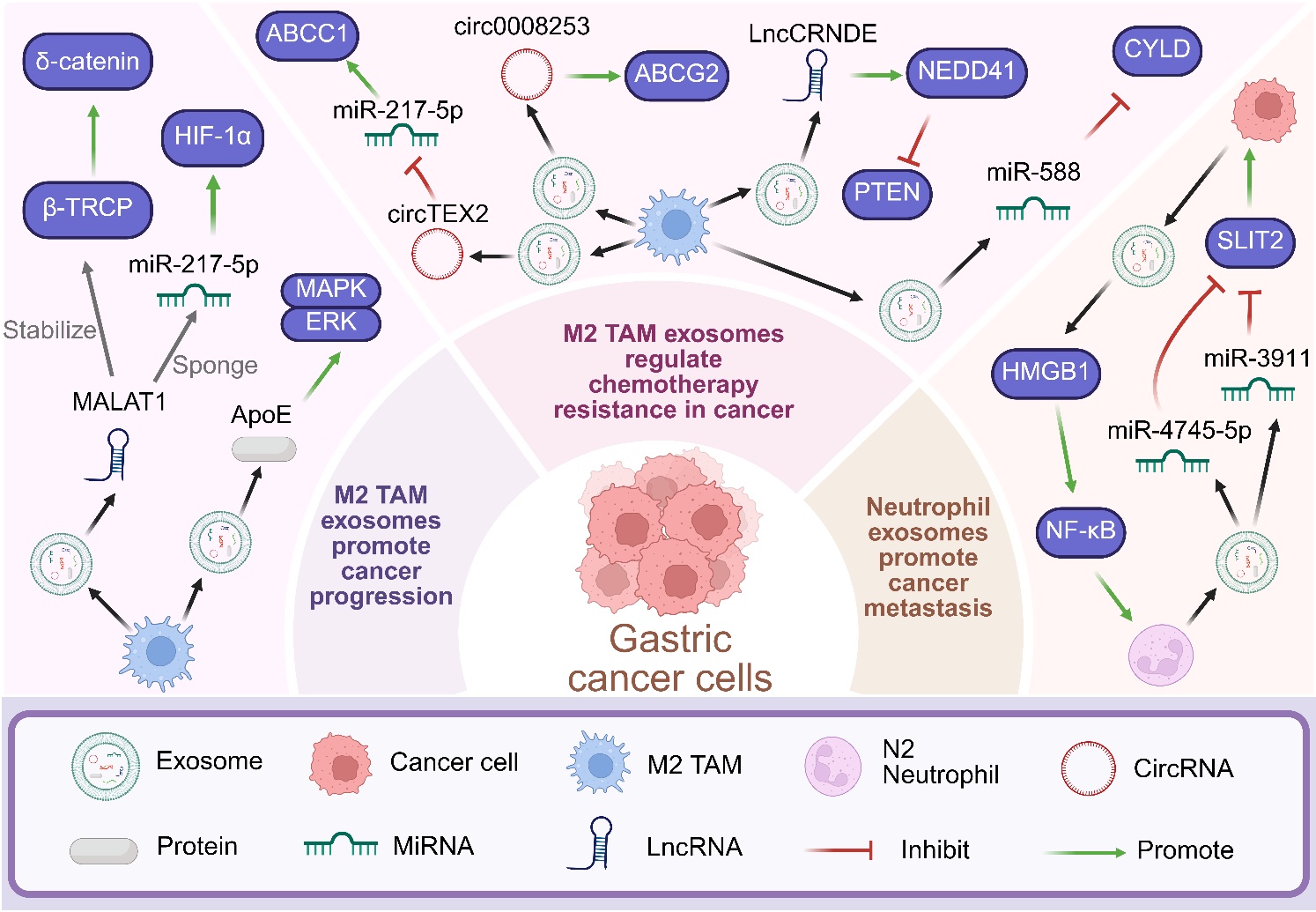
**
